# The Role of Psychological Pricing Strategy in Customer Purchasing Behavior

**DOI:** 10.12688/f1000research.177273.1

**Published:** 2026-06-01

**Authors:** Asst. Prof. Dr. Sawsan Ibrahim Rajab, MOHAMMED SATWAN AMHAMMED

**Affiliations:** 1 Northern Technical University, Mosul, Nineveh Governorate, Iraq

**Keywords:** Keywords: Psychological pricing, fractional prices, price separators, price font size, consumer purchasing behavior, Al-Salam chain stores.

## Abstract

This study aimed to clarify the role of psychological pricing strategies, specifically the strategies of partial pricing, price increments, price line size, and price line color, in guiding the purchasing behavior of customers of Al-Salam chain stores in Kirkuk Governorate, as represented by its combined dimensions (cognitive response, emotional response, and purchase). The research problem stemmed from the lack of awareness among the management of Al-Salam chain stores in Kirkuk Governorate regarding the impact of these strategies on consumer purchasing behavior. Psychological pricing is supposed to be a tool for maximizing sales and acquiring the largest possible number of customers. Therefore, to achieve the objective and address the research problem, two main hypotheses were adopted, seeking to determine the role of psychological pricing strategies, represented by their dimensions (partial pricing, price increments, price line size, and price line color), in guiding consumer purchasing behavior. To collect and analyze data, a questionnaire was designed in accordance with the study’s objective and distributed to 400 customers of Al-Salam chain stores in Kirkuk Governorate. Of these, (288) questionnaires were valid for statistical analysis, and their data formed the basis for reaching a set of results, which proved the existence of a strong correlation and influence of psychological pricing at the micro level, represented by (sub-prices, price period, price line size, and price line color), and at the macro level in directing the customer’s purchasing behavior with its aggregated dimensions, represented by (cognitive response, emotional response, and purchase).

## Introduction

Marketing is considered one of the fastest-developing sciences due to the changes it continuously undergoes in form, time, information, economy, and technology. These changes in the economic arena play an important role in bringing about fundamental transformations in marketing science, and all these developments have been reflected in the customer and their decisions. Accordingly, research and studies have increasingly focused on understanding the influence on consumer purchasing behavior and how to direct it toward available products, as the customer is the starting and ending point of the marketing process.

Therefore, organizations have sought to adopt all strategies that attract customers, satisfy their desires, and meet their needs, as well as to raise their sales levels, profits, and market share. This can only be achieved by influencing the consumer’s purchasing behavior, prompting them to make purchase decisions without deep reflection.

From here, the connection between marketing and psychology began to take shape, further confirmed by the famous saying: “Where there is a human being, there is psychology.” Thus, psychological pricing emerged as a type of psychological strategy focused on stimulating emotions and feelings and relying solely on the psychological aspect to attract customers and influence their purchasing behavior. Accordingly, this study came as a reflection of this idea with the aim of clarifying the concept of psychological pricing and presenting some of its strategies to demonstrate the importance of this type of pricing, which can influence consumer behavior and strengthen their purchasing decisions.

## Chapter One: Research methodology

### First: Research problem

The rapid global changes resulting from the manifestations of globalization have led to the opening and liberalization of markets and intensified competition among organizations, giving consumers the opportunity to choose among the different products offered by these organizations. To attract customers, ensure their loyalty, and sell their products, organizations have adopted various strategies, one of which is psychological pricing. This strategy intertwines the relationship between pricing, as one element of the marketing mix, and psychology. All organizations seek to understand customers’ needs, desires, and purchasing behavior. To solve this challenge, marketers have integrated psychological concepts into marketing strategies in general, and pricing strategies in particular, to address customers more effectively, thereby attracting and motivating them to purchase and achieving the organization’s goals of increasing sales, profits, and market share.

Through researchers’ visits to Al-Salam chain stores in Kirkuk Governorate, it was found that although the chain’s management adopts psychological pricing, it is not fully aware of how this strategy influences consumer purchasing behavior. Psychological pricing is supposed to be their tool to achieve maximum sales and gain as many customers as possible. Hence, the study problem was identified through the following central question:

“Is there an effect of psychological pricing on consumer purchasing behavior?”

From this main question, several sub-questions arise:

1. Is there an correlation and effect of fractional prices on consumer purchasing behavior?
2. Is there an correlation and effect of price separators on consumer purchasing behavior?
3. Is there an correlation and effect of price font size on consumer purchasing behavior?
4. Is there an correlation and effect of price font color on consumer purchasing behavior?

### Second: Importance of the study

The importance of this study stems from its two variables: psychological pricing is an important strategy for achieving organizational goals, while consumer purchasing behavior is one of the key aspects all organizations seek to study, analyze, and direct toward their products. In addition:

1. Drawing the attention of Al-Salam chain store management in Kirkuk to the importance of psychological pricing strategy in achieving their goals and its impact on consumer purchasing behavior.
2. Pricing is the only element that generates revenue for the organization and also serves as its direct link with customers. Accordingly, adopting an appropriate pricing strategy is a tool to attract and motivate customers to purchase.
3. Raising customers’ awareness of the pricing strategies used, including psychological pricing, and their impact on their purchasing behavior.

### Third: Research objectives

In light of the research problem and its importance, the study aims to achieve the following objectives:

1. Provide a theoretical framework defining both psychological pricing strategy and consumer purchasing behavior.
2. Identify the nature of the relationship (in terms of correlation and influence) between psychological pricing strategy (in its combined and individual dimensions) and consumer purchasing behavior.
3. Arrive at a set of findings and recommendations to improve the marketing mechanisms of business organizations in the local market, with the goal of maintaining their market share and attracting more customers.
4. Emphasize the role of applying psychological pricing strategy to increase organizations’ sales volume and achieving profits.

### Fourth: Study framework

Addressing the research problem in light of its theoretical framework and field implications requires designing a conceptual model, as shown in Figure 1, which illustrates the relationship and influence between psychological pricing strategy and consumer purchasing behavior.

**Figure 1.**
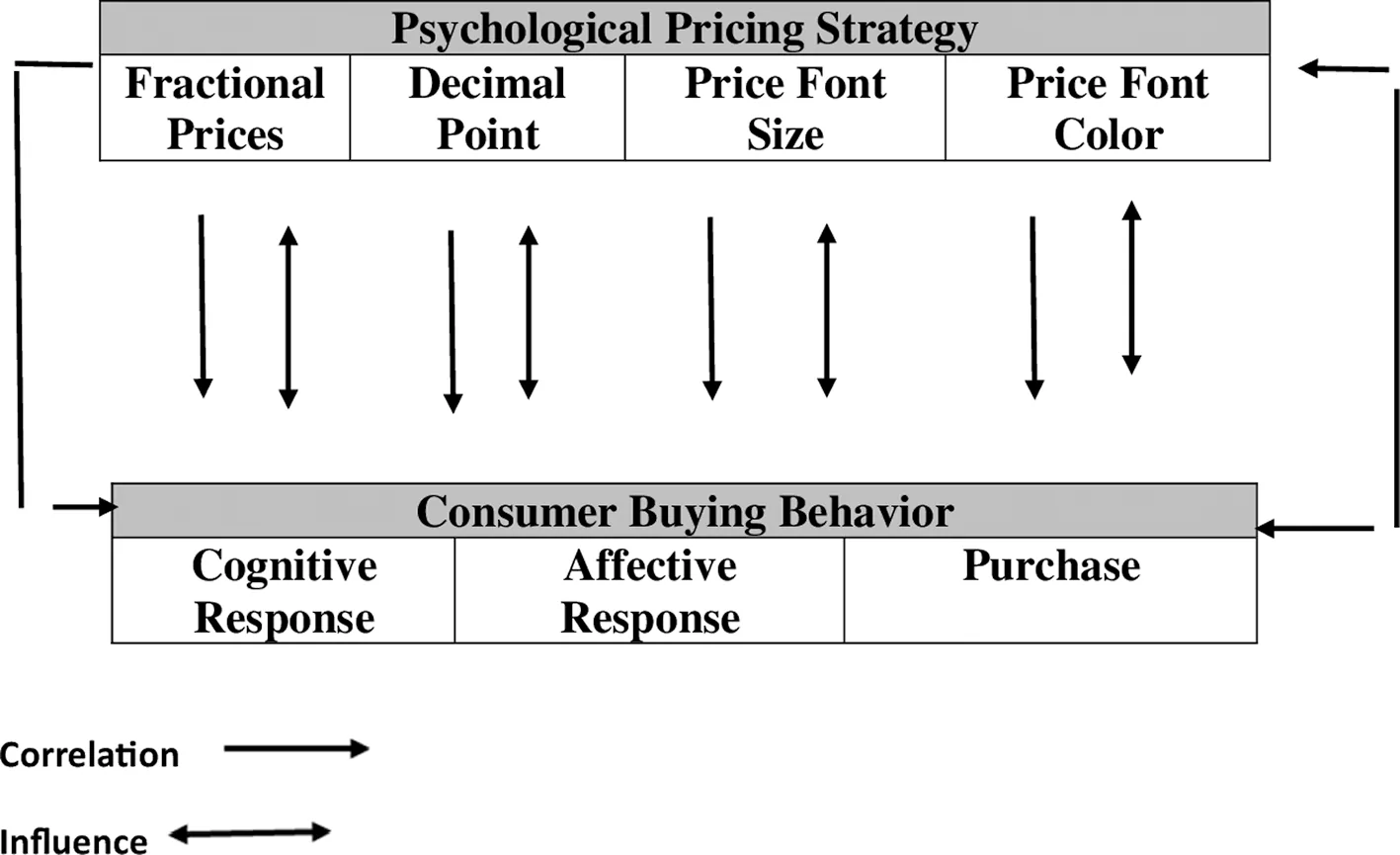
Study framework.

### Fifth: Study hypothesis

The study is based on two main hypotheses, from which sub-hypotheses branch out as follows:

### Main hypothesis one

There is a statistically significant correlation between the psychological pricing strategy (at the overall level) and customer purchasing behavior (at the overall level) among the customers of the researched chain. From this emerge the following sub-hypotheses:

• **Sub-hypothesis One**: There is a statistically significant correlation between the psychological pricing strategy, represented by (fractional prices), and customer purchasing behavior (at the overall level) among the customers of the researched chain.
• **Sub-hypothesis Two**: There is a statistically significant correlation between the psychological pricing strategy, represented by (price separator), and customer purchasing behavior (at the overall level) among the customers of the researched chain.
• **Sub-hypothesis Three**: There is a statistically significant correlation between the psychological pricing strategy, represented by (price line size), and customer purchasing behavior (at the overall level) among the customers of the researched chain.
• **Sub-hypothesis Four**: There is a statistically significant correlation between the psychological pricing strategy, represented by (price line color), and customer purchasing behavior (at the overall level) among the customers of the researched chain.

### Main hypothesis two

There is a statistically significant positive effect of the psychological pricing strategy (at the overall level) on customer purchasing behavior (at the overall level) among the customers of the researched chain. From this emerge the following sub-hypotheses:

• **Sub-hypothesis One**: There is a statistically significant effect of the psychological pricing strategy, represented by (fractional price strategy), on customer purchasing behavior (at the overall level) among the customers of the researched chain.
• **Sub-hypothesis Two**: There is a statistically significant effect of the psychological pricing strategy, represented by (price separator strategy), on customer purchasing behavior (at the overall level) among the customers of the researched chain.
• **Sub-hypothesis Three**: There is a statistically significant effect of the psychological pricing strategy, represented by (price line size), on customer purchasing behavior (at the overall level) among the customers of the researched chain.
• **Sub-hypothesis Four**: There is a statistically significant effect of the psychological pricing strategy, represented by (price line color), on customer purchasing behavior (at the overall level) among the customers of the researched chain.

### Sixth: Study methodology and data collection methods

The study adopted a descriptive-analytical approach. Its theoretical framework was built based on available relevant references, while its field component relied on a questionnaire as the primary tool for collecting and tracking data and transforming it into information. From this information, the researchers were able to deduce aspects that covered the reality of the variables adopted in the study plan. The questionnaire was designed for this purpose and used as a source for collecting data, which was then analyzed, yielding results that contributed to achieving the study’s objectives. The questionnaire was designed with clear and understandable items for the surveyed sample, and it was measurable so that the study variables could be measured in terms of structure and wording. A five-point Likert scale was used to measure the weight of the respondents’ answers. The items were (Strongly Agree, Agree, Neutral, Disagree, Strongly Disagree), with weights of (1, 2, 3, 4, 5) respectively. The tendency of respondents’ answers towards (Strongly Agree, Agree) indicates a state of agreement that reflects the availability of the variables’ dimensions, while (Neutral) indicates a state of agreement. To reflect a state of moderation, while “I disagree” and “I strongly disagree” express a state of disagreement. The responses of the chain’s customers in the research sample were analyzed using statistical tools, enabling the study to arrive at results that allowed it to offer a set of conclusions and suggestions that it hopes will be valuable to the chain’s management in its marketing efforts.

### Seventh: Study boundaries

• **Time Boundaries**: The study aims to explore customers’ opinions during the specified period from 2/1/2025 to 4/1/2025.
• **Spatial Boundaries**: The study was limited to the branches of Al-Salam retail chain stores inside Kirkuk Governorate, totaling (4) branches, which served as the field of the study.
• **Human Boundaries**: A total of (400) questionnaires were distributed individuals from the customers of Al Salam chain of stores who expressed their agreement and willingness to cooperate and answer the questionnaire, and (288) valid questionnaires were retrieved for statistical analysis.

### Eighth: Description of the study field, population, and sample

**A: Introductory Overview of Al-Salam Retail Chain in Kirkuk Governorate**

Al-Salam retail chain was established in Kirkuk Governorate in (2003). It has (10) branches, (4) of which are inside Kirkuk city, while the rest are distributed across the main districts of the governorate. These branches offer a wide variety of branded products from different origins, all of which relate to the essential needs of Iraqi families. A large number of customers frequent these stores due to the availability of diverse product lines that meet their needs and satisfy their desires.

All stores are managed by a Board of Directors that focuses on developing business plans and making the necessary decisions for the chain’s growth, sustainability, and profitability, thereby ensuring its continuity and strengthening its competitive position in the local Kirkuk market compared to other retail chains operating in the same governorate.

The stores employ a sufficient number of experienced and qualified sales staff who provide various services to facilitate customer access to products, in addition to offering necessary information about any displayed product when needed.

**B: Description of the Study Population and Sample**

The study population was defined as the customers shopping at the (4) branches of Al-Salam commercial chain in central Kirkuk. For the purpose of distributing the questionnaire to shoppers, four employees were appointed and distributed across the four branches. Their task was to receive customers, explain the purpose of the questionnaire, and encourage them to participate by answering its items. However, they faced difficulty in convincing many shoppers because the majority of them did not want to participate by expressing their opinions, or because they did not understand their role in building future pricing strategies. Some made excuses by saying that they did not have enough time to participate. Only (400) questionnaires were distributed to a diverse sample questionnaires were distributed among a diverse sample of customers shopping for different products of various brands and origins displayed by the chain, those who agreed to participate by stating their opinions on the questionnaire items. (288) valid questionnaires were retrieved for statistical analysis. Table 1 shows the demographic data of the sample studied.

**Table 1.** Demographic data for the sample group.

#	Demographic data	Categories	Number	%
1	Gender	Female	167	57
		Mal	121	42
2	Age group	(18-28) years	43	15
		(29–39) years	92	32
		(40–50) years	97	34
		(51) years and over	56	19
3	Social status	Married	193	67
		Single	95	33

Source: Researchers’ calculations based on AMOS V24 results N = 288.

## Chapter One: Theoretical framework

### Section One: Psychological pricing

**First: Psychological Pricing – Concept, Importance, and Objectives**

Psychological pricing is considered an important concept in marketing and business management, playing a crucial role in determining product prices. Psychological pricing refers to a “policy that relies primarily on price to influence the psychological state of the customer, where the price is adjusted according to customer type. For some, high prices mean something, while others may be attracted to anything advertised at a low price”. ^1^ It is also defined as “the process of setting prices based on customer lists, where it depends on their emotional responses, subjective evaluations, and feelings toward specific purchases”. ^2^ According to ^3^ it is “policies that rely on influencing the customer’s psychological state to drive them to make a purchase decision as a result of an emotional reaction rather than depending on rational thought in purchasing.”

Accordingly, psychological pricing can be defined as “a pricing strategy that uses specific methods to influence customer perceptions and behavior, exploiting and employing their emotional responses to certain price points, making products appear more attractive, thereby generating a desire to purchase.”

Businesses adopt psychological pricing within their marketing offers due to its importance, which can be clarified as follows ^4^:

1. Enables the organization to identify what customers like in its products. During marketing campaigns, they focus on appealing to customer emotions by knowing their preferences and emphasizing what benefits them.
2. Maintain continuous communication with customers to build closeness.
3. Use multiple methods in product marketing based on marketing mix elements, all adjusted to psychological influences.
4. Attract customers by focusing on the emotional and psychological side.
5. Present marketing offers to customers in an attractive way.
6. Study the psychological impact of surrounding environmental influences on customers.
7. Identify what might prevent customers from making purchasing decisions, which affects marketing activities and operations.
8. Help improve marketing campaigns and strategies for more effective influence on both customers and the purchasing process.
9. Study customer psychology, decision-making, and preferences among various brands.

The use of psychological pricing as part of promotional tools helps organizations achieve several goals, including ^5^:

1. Simplifying the purchase decision-making process for the customer and identifying cost-conscious customers, as this is one of their main weaknesses. With the displayed price, they spend less time thinking, enabling them to make a purchase decision quickly as an opportunity to seize.
2. Designing discount systems with distinctive impact, influencing the customer’s emotional desire to purchase.
3. Making the customer feel intelligent and successful, as though they got a good deal and outsmarted the general pricing system followed by competing organizations offering similar products at higher prices. ^6^
4. Helping the organization determine the cost of products it intends to sell, thereby enabling it to set a more effective marketing strategy for its products.
5. Enhancing sales, raising levels, and enabling a wider segment of customers to purchase these products.
6. Building a good reputation and a positive brand image in the customer’s mind, positioning the organization as one that wants everyone to have access to good products at reasonable prices that meet desires, satisfy needs, and make the customer feel they achieved the best purchase. ^7^

**Second: Psychological Pricing Strategies**

There are multiple psychological pricing strategies, and organizations choose the strategy that best suits their internal and external conditions. They may also reconsider any psychological pricing strategy currently being followed, based on changes and updates in the internal and external marketing environment, and adopt another strategy more aligned with these changes ^10, 8 9^ identified psychological pricing strategies as (Fractional Prices, Comma Effect, Font Size Effect, Price Anchoring), ^11, 12^ and agree with him in identifying the same strategies that can be adopted within psychological pricing, while ^13^ identified psychological pricing strategies as (Fractional Prices, Price Interval, Hundred Rule for Discounts, Word Free, Font Size When Writing Old and New Price, Red Color and its Effect on Writing Price, Effect of Descending Order of Prices).

Based on the above, we see that there is agreement on identifying some psychological pricing strategies that the organization can adopt in its marketing program, certainly in accordance with the requirements and challenges of its internal and external environment. For the purposes of this research, we will address the strategies of (fractional pricing, price interval, price line size, and price line color), as follows ^14^:

**1- Fractional Pricing**

According to this strategy, prices are called “attractive prices.” Through this strategy, the organization attempts to influence the customer psychologically by suggesting that the product is lower in price because it is slightly less than a round number. ^14^ The organization uses fractions of the currency unit, such as stating that the selling price of a unit is $3.99 instead of $4. This fractional number gives the customer the impression that prices have been reduced and set with great precision. ^15^

**2- Price Punctuation**

According to this strategy, the organization believes that if it places a punctuation mark, the customer will perceive the price differently. For example, when a product is priced as (1500$), the customer sees it differently compared to (1.500$). Although any rational person knows they are the same amount, this small punctuation mark creates a strong difference in the customer’s perception of the price. Most customers perceive prices written with punctuation as higher than those without, as people interpret numbers visually, which influences their purchase decisions. ^16^

**3- Price Font Size**

Reducing the font size in which the price is written on the package directly, or on the tag placed on the product, is one of the most effective strategies for increasing sales. Products with smaller-sized price fonts are much more likely to be purchased than those with large-sized price fonts. This strategy is based on the idea that smaller font sizes are psychologically associated with lower prices. In the customer’s mind, font size is linked with value—smaller font suggests a smaller price, and vice versa. ^17^

**4- Price Font Color**

The color of the price font significantly affects customer behavior and purchase decisions. The colors used to write prices on displayed products can trigger different feelings and emotions, influencing how customers interact with prices and whether they will purchase the product. Red, for example, is often used by marketers on discount tags to grab attention and stimulate the desire to buy, while blue may be used for luxury products to suggest quality and trust. ^18^

## Axis Two: Customer buying behavior

### First: The concept and importance of customer buying behavior

Understanding customer behavior is a crucial stage in the marketing process. This process, driven by the need to meet customer demands, is by no means easy. Customers, for example, may express interest in buying a product but not actually purchase it, ^19^ or they may not know exactly what they want to buy. They may also make their decisions at the last minute depending on their circumstances. Therefore, marketing management must analyze customer needs and understand how they perceive things—this means paying attention to their buying behavior. ^20^

**Customer buying behavior has been defined as:**

• “The set of actions a customer undertakes when choosing a product available in the market—the feelings and thoughts the customer relies on before or during the purchase of any product. The final outcome of this behavior is the customer’s ultimate decision regarding product selection, brand preference, choice of sales outlet, timing of purchase, and frequency of purchase”. ^21^
• “The mental processes and behavioral actions the customer undertakes when intending to buy a product, as they search for a product that achieves the desired level of satisfaction for their needs and desires and aligns with their expectations”. ^22^
• ^23^defines it as “the behaviors or stages an individual goes through when making a purchase decision for a product in order to satisfy their needs. Brand owners study customer behavior to design strategies and advertising campaigns directed at customers”.

The importance of studying customer buying behavior lies in the fact that it achieves several objectives for both the customer and the organization, as follows ^24^:

1. Helps the organization design products in ways that ensure customer acceptance, thereby increasing acquisition rates. The more the organization understands its customers, the better it can serve and satisfy their needs and achieve its goals.
2. Enables the organization to discover new marketing opportunities by researching unmet and emerging customer needs and investing in them to diversify its products, enhance competitiveness, and increase market share.
3. Understanding customer behavior and their purchasing power helps the organization set pricing policies. A successful organization is one that offers products that satisfy customer desires within their purchasing capacity.
4. Enables the organization to evaluate its marketing performance and identify strengths and weaknesses by knowing customers’ opinions about its products.
5. Helps determine distribution channels for its products—whether by relying on its own outlets with a focus on personal selling and sales representatives for direct customer interaction, or by relying on intermediaries and agents (wholesalers and retailers) for indirect interaction. ^25^
6. Assists the organization in designing promotional policies. By understanding customer tastes and preferences, marketing management can determine a suitable promotional mix aimed at influencing and persuading customers to buy its products. ^26^

### Second: Dimensions of customer buying behavior

Customer purchasing behavior can be measured through several dimensions, which will also be adopted in this study due to their consistency with the data. These dimensions include ^27^:

• **Perceptual Response**: The starting point of any customer behavior is an unfulfilled need. Marketing programs show the importance of a product, as well as its features and benefits. Once the customer recognizes the need for a product, they begin to gather information about it to determine if it meets their need. ^28^ Customers rely on multiple sources to support what they already know, such as their social environment, which provides information that organizations should use in marketing programs to differentiate their products from competitors, giving customers knowledge that makes them prefer the organization’s product. ^29^
• **Emotional Response**: Emotional response reflects the customer’s feelings and emotions toward the purchase subject. If the customer feels liking or disliking, they may develop positive or negative attitudes toward the product. The emotional relationship the customer forms with the product is complex, involving multiple behavioral levels and a mix of conscious and unconscious internal and external reactions. Product evaluation is influenced by logical judgments (e.g., performance, ease of use, necessity of ownership) as well as subjective ones (e.g., lack of rational justification but strong personal desire to own it). ^30^
• **Purchase**: The buying decision process is defined as the procedure through which customers collect and analyze information and choose among product or organizational alternatives. It also consists of coordinated measures taken to satisfy needs. ^31^ Many situational factors affect the purchase decision, such as time pressure. For example, limited time may lead to a hasty decision without sufficient information to evaluate alternatives, possibly resulting in customer dissatisfaction post-purchase. ^32^

### Section Three: Field framework

In this section, the study hypotheses will be tested by determining and analyzing the correlation and impact relationship between psychological pricing strategies and customer buying behavior, as follows:

**First: The Relationship Between Psychological Pricing Strategies and Customer Buying Behavior**

• **Main Hypothesis 1**: There is a statistically significant correlation between psychological pricing strategies (at the overall level) and customer buying behavior (at the overall level) among the customers of the studied chain
• The results of Table 2 show the existence of a positive, statistically significant correlation between the independent variable (psychological pricing strategy) and the dependent variable (customer purchasing behavior), as indicated by the overall correlation coefficient of (0.91). This reflects a strong positive relationship between the study’s variables, based on the probability value (P-value), which was found to be (0.002), less than (0.05). Moreover, the similarity of the signs of both the Upper and Lower limits of the 95% Confidence Interval at the significance level (0.005) further supports this This relationship points to psychological pricing as one of the important strategies that enables the research chain to offer its products at attractive prices that encourage customers to buy. Accordingly, the first main hypothesis is accepted, which states:
• “There is a statistically significant correlation between the psychological pricing strategy (at the overall level) and customer purchasing behavior (at the overall level) among the customers of the studied chain.”

**Table 2.** Correlation between psychological pricing strategy and consumer buying behavior.

First variable	Direction of relationship	Second variable	Correlation value	95% Confidence interval		P - value
				Upper	Lower	
Psychological Pricing Strategy	→←	Consumer Buying Behavior	0.91	0.951	0.845	0.002

Source: Researchers’ calculations based on AMOS V24 results (α ≤ 0.05, n = 288).

**2- The sub-hypotheses derived from the first main hypothesis**

Based on the data in Table 3, the following becomes clear:

• **First Sub-Hypothesis **:
• There is a statistically significant positive correlation between the psychological pricing strategy, represented by fractional prices, and customer purchasing behavior (at the overall level) among the customers of the studied chain.
• A statistically significant positive relationship exists between fractional prices and purchasing behavior, as indicated by the correlation coefficient of (0.91). This relationship is significant based on the P-value, which was (0.003), less than (0.05). Moreover, the signs of both the Upper and Lower limits of the (95% Confidence Interval) at the significance level (0.05) were similar. This relationship suggests that adopting fractional prices motivates customers and directs their purchasing behavior, as they perceive it as an opportunity to achieve savings when buying. Accordingly, the first sub-hypothesis derived from the first main hypothesis is accepted.
• **Second Sub-Hypothesis:**
• There is a statistically significant correlation between the psychological pricing strategy, represented by price separators, and customer purchasing behavior (at the overall level) among the customers of the studied chain.
• A statistically significant positive relationship exists between price separators and purchasing behavior, as indicated by the correlation coefficient of (0.79). This relationship is significant based on the P-value, which was (0.002), less than (0.05). Moreover, the signs of both the Upper and Lower limits of the (95% Confidence Interval) at the significance level (0.05) were similar. This relationship shows that price separators play a role in guiding customer purchasing behavior, encouraging them to buy more products. Therefore, the second sub-hypothesis derived from the first main hypothesis is accepted.
• **Third Sub-Hypothesis:**
• There is a statistically significant correlation between the psychological pricing strategy, represented by price font size, and customer purchasing behavior (at the overall level) among the customers of the studied chain.
• A statistically significant positive relationship exists between price font size and purchasing behavior, as indicated by the correlation coefficient of (0.82). This relationship is significant based on the P-value, which was (0.003), less than (0.05). Moreover, the signs of both the Upper and Lower limits of the (95% Confidence Interval) at the significance level (0.05) were similar. This result indicates that the size of the font in which the price is written on displayed products plays a role in guiding customer behavior toward purchasing, as smaller font sizes are perceived to be associated with lower prices. Based on this, the third sub-hypothesis derived from the first main hypothesis is accepted.
• **Fourth Sub-Hypothesis:**
• There is a statistically significant correlation between the psychological pricing strategy, represented by price font color, and customer purchasing behavior (at the overall level) among the customers of the studied chain.
• A statistically significant positive relationship exists between price font color and purchasing behavior, as indicated by the correlation coefficient of (0.68). This relationship is significant based on the P-value, which was (0.002), less than (0.05). Moreover, the signs of both the Upper and Lower limits of the (95% Confidence Interval) at the significance level (0.05) were similar. This result shows that using colors in writing prices, or highlighting a specific feature of the product, captures the customer’s attention and encourages them to purchase. Accordingly, the fourth sub-hypothesis derived from the first main hypothesis is accepted.

**Second: The Effect of Psychological Pricing Strategy on Customer Purchasing Behavior**

**Table 3.** Correlation between dimensions of psychological pricing strategy and consumer buying behavior.

First variable	Direction of relationship	Second variable	Correlation value	95% Confidence interval		P – value
				Upper	Lower	
Psychological Pricing Strategy	→←	Consumer Buying Behavior	0.91	0.956	0.843	0.003
Fractional Prices	→←		0.79	0.884	0.683	0.002
Decimal Prices	→←		0.82	0.887	0.720	0.003
Price Font Size	→←		0.68	0.791	0.555	0.002
Price Font Color	→←		0.91	0.956	0.843	0.003

Source: Researchers’ calculations based on AMOS V24 results AMOS V24 (α ≤ 0.05, n = 288).

**1- Second Main Hypothesis:**

There is a statistically significant positive effect of the psychological pricing strategy (at the overall level) on customer purchasing behavior (at the overall level) among the customers of the studied chain.

Table 4: The Effect of Psychological Pricing Strategy on Customer Purchasing Behavior.

**Table 4.** Effect of psychological pricing strategy on consumer buying behavior.

Independent variable	Direction of effect	Dependent variable	Regression coefficient estimate (β)	Standard error of β (Se.(β))	R ^2^ (Coefficient of determination)	Critical ratio (C.R.)	P - value
Psychological Pricing Strategy	-->	Consumer Buying Behavior	0.907	0.028	0.82	32.392	0.002

Critical t-value: (t Tab = 1.96).

Source: Researchers’ calculations based on AMOS V24 results (α ≤ 0.05, n = 288).

The results shown in Table 3 indicate the existence of a substantial effect of the independent variable, “psychological pricing strategy” (at the overall level), on the dependent variable, “customer purchasing behavior” (at the overall level). This is evidenced by the value of (β), which reached (0.907). This effect is statistically significant as indicated by the P-value of (0.002), which is less than (0.05). The same result is also confirmed by the (C.R.) value, which reached (32.392), exceeding the tabulated t-value (t Tab) of (1.96).

Furthermore, the value of (R ^2^), which amounted to (0.82), indicates a significant effect of the independent variable on the dependent variable. This means that the independent variable (psychological pricing strategy) explains (82%) of the variance in the dependent variable (customer purchasing behavior), while the remaining percentage is attributed to factors beyond the scope of this study. This result shows that customer purchasing behavior is clearly influenced by psychological pricing strategies, thus motivating them to buy. Accordingly, the second main hypothesis is accepted.

**2- The sub-hypotheses derived from the second main hypothesis**

Based on the data presented in Table 5, the following becomes clear:

**Table 5.** The existence of the dimensions of the psychological pricing strategy in customer purchasing behavior.

Independent variable	Direction of effect	Dependent variable	Regression coefficient estimate (β)	Standardized Regression Weight (SRW)	Standard error of β (Se.(β))	Coefficient of determination (R ^2^)	Critical ratio (C.R.)	P-value
Fractional Prices	-->	Consumer Buying Behavior	0.909	1.091	0.135	0.87	3.117	0.003
Decimal Prices	-->		0.797	0.831	0.106		2.920	0.002
Price Font Size	-->		0.685	0.601	0.079		7.607	0.002
Price Font Color	-->		0.824	0.829	0.092		9.010	0.003

Source: Researcher’s calculations based on AMOS V24 results (α ≤ 0.05, n = 288) Critical t-value: t Tab = 1.96.

**• First Sub-Hypothesis:**

There is a statistically significant effect between the psychological pricing strategy, represented by (fractional pricing), and customer purchasing behavior (at the aggregate level) among the customers of the studied chain.

There is an effect of the fractional pricing dimension on customer purchasing behavior, as indicated by the value of (β), which reached (1.091). This effect is statistically significant, with a p-value of (0.003), which is less than (0.05). The same result is confirmed by the C.R. value, which reached (3.117), greater than the tabulated t-value (1.96), These results show that customers expect to achieve greater savings when purchasing products priced using fractional pricing. This leads us to accept the first sub-hypothesis derived from the second main hypothesis.

**• Second Sub-Hypothesis:**

There is a statistically significant effect between the psychological pricing strategy, represented by (the price separator), and customer purchasing behavior (at the aggregate level) among the customers of the studied chain.

There is an effect of the price separator dimension on customer purchasing behavior, as indicated by the value of (β), which reached (0.831). This effect is statistically significant, with a p-value of (0.002), which is less than (0.05). The same result is confirmed by the C.R. value, which reached (2.920), greater than the tabulated t-value (1.96), These results show that the customer feels that the prices determined by using the comma represent the true price of the product and that their purchase of this product aligns with their personal capabilities and desires. This leads us to accept the second sub-hypothesis derived from the second main hypothesis.

**• Third Sub-Hypothesis:**

There is a statistically significant effect between the psychological pricing strategy, represented by (price font size), and customer purchasing behavior (at the aggregate level) among the customers of the studied chain.

There is an effect of the price font size dimension on customer purchasing behavior, as indicated by the value of (β), which reached (0.601). This effect is statistically significant, with a p-value of (0.002), which is less than (0.05). The same result is confirmed by the C.R. value, which reached (7.607), greater than the tabulated t-value (1.96), These results show that the font size used in product pricing attracts attention. When the new price of a product is displayed in a font size different from the size used for the old price, it ensures that the customer will notice the price, and this can stimulate their desire to buy due to the difference between the old and new prices Thus, the customer has made a good deal when purchasing the product. This leads us to accept the third sub-hypothesis derived from the second main hypothesis.

**• Fourth Sub-Hypothesis:**

There is a statistically significant effect between the psychological pricing strategy, represented by (price font color), and customer purchasing behavior (at the aggregate level) among the customers of the studied chain.

There is an effect of the price font color dimension on customer purchasing behavior, as indicated by the value of (β), which reached (0.829). This effect is statistically significant, with a p-value of (0.003), which is less than (0.05). The same result is confirmed by the C.R. value, which reached (9.010), greater than the tabulated t-value (1.96), These results show that colors have clear effects on customer decisions. Adopting a different color for the new price than the old price color draws the customer’s attention to the fact that there are price reductions and the purchase process will be profitable for him. Also, adopting a specific color for pricing some products, such as luxury or distinctive products, makes the customer feel the value of these products and motivates him to buy. This leads us to accept the fourth sub-hypothesis derived from the second main hypothesis.

## Chapter Four: Conclusions and recommendations

### First: Conclusions

1. Customer purchasing behavior in its dimensions is of great interest to the management of Al-Salam retail chain in Kirkuk, as the customer is the foundation for the chain’s survival, growth, and continuity. Moreover, the customer is the focus of all the marketing activities adopted by the chain, and all its efforts are directed at satisfying the customer and persuading them to purchase.
2. There is a significant positive correlation between psychological pricing (with its dimensions: fractional pricing, price separator, price font size, and price font color) and customer purchasing behavior (with its dimensions: cognitive response, emotional response, and purchasing) in the studied chain.
3. There is a significant positive correlation between each individual dimension of psychological pricing (fractional pricing, price separator, price font size, and price font color) and customer purchasing behavior (cognitive response, emotional response, and purchasing) in the studied chain.
4. There is a significant positive effect of psychological pricing, both collectively and individually (fractional pricing, price separator, price font size, and price font color), on customer purchasing behavior (cognitive response, emotional response, and purchasing) in the studied chain.
5. Using fractional numbers in displaying product prices leads customers to believe that the pricing process is precise and objective.
6. The use of a price separator and writing the numbers after the comma in a different font size and color is more influential and attention-grabbing than a price without a separator for the same product, making customers feel that the products are cheaper.
7. Writing the price in a clear, large, or different font style draws customers’ attention to products and generates in them the desire to make a purchase.

### Second: Recommendations

In light of the study results and conclusions, it is appropriate to present the following recommendations:

1. The management of Al-Salam retail chain should pay greater attention to psychological pricing, as it is one of the most important pricing strategies that primarily address the psychological side of the customer, driving them to make a purchase decision based more on emotional and psychological reaction than logical reasoning.
2. The management of the studied retail chain should recognize that psychological pricing strategy elevates the value of the product in customers’ perception by instilling positive feelings when they see the products and their prices; since customers purchase more with emotions than through deep rational thought.
3. Using psychological pricing as a pricing strategy by Al-Salam retail chain management has a significant impact on increasing sales, and consequently increasing both profits and market share.
4. The management of Al-Salam retail chain should give greater attention to psychology and its influence on marketing in general and pricing in particular, as it is a science based on marketing strategies that rely on purely psychological studies of the customer. This enables the chain to attract more customers and influence their purchasing behavior.
5. The chain’s management should pay more attention to the dimensions of psychological pricing strategy to enhance its role in achieving better results regarding guiding customer purchasing behavior.
6. The chain’s management should be encouraged to focus on broader dimensions of customer purchasing behavior, studying and employing them as a competitive tool against rival stores.
7. The management of Al-Salam retail chain should seek assistance from psychology experts, as this will bring them closer to the customer and make it easier to understand their needs and desires, thereby providing them with the best offerings. This should be based on psychological research tailored to the local customer environment in order to achieve the best possible results.
8. The studied chain’s management should conduct ongoing study of the relationship between the dimensions of psychological pricing strategy and customer purchasing behavior.
9. Employees of Al-Salam retail chain should be trained in customer interaction techniques and how to motivate and encourage customers to purchase.

### Limitations and future research

Although the study contributed to achieving its stated objectives, there are several limitations that must be acknowledged. These limitations are as follows:

1. Data was collected from customers of the studied retail chain. Although the number of participants reached 400, this number is insufficient given the large customer base of this chain. The refusal to participate stemmed from several reasons, including apprehension about participating, time constraints, and a lack of awareness of the importance of scientific research and its role in improving marketing performance. Therefore, generalizing the results is certainly appropriate for the studied retail chain, but it is essential to expand the study to include other retail centers if they wish to utilize the findings in their marketing operations and activities.

2. Verbal consent was obtained from customers who participated in answering the questionnaire items. This was due to their reluctance to write their names and sign any other document, whether it be the questionnaire or otherwise, with their name and legal information, fearing its potential misuse for purposes that could harm them in the future. Therefore, verbal consent was sufficient to ensure cooperation and completion of the questionnaire.

3. The study relied on self-reported measures, which may introduce bias into the responses or influence social desires that could affect the accuracy of participants’ answers.

4. The research focused primarily on psychological pricing strategies and their impact on customer purchasing behavior. It did not address other pricing strategies or factors that may influence customer purchasing behavior.

5. Although many studies have examined customer purchasing behavior, several new factors have emerged as a result of technological advancements and modern communication methods. These factors should be added to those mentioned previously in this study, and further research is needed.

6. Research should aim to study and analyze the causal relationships that can influence customer purchasing behavior through the application of more comprehensive psychological pricing strategies across diverse or specific customer groups and in different social environments. This will help identify causal pathways and clarify the nature of the correlation and influence between them.

7. Studies should employ a mediating or moderating variable between psychological pricing strategies as the independent variable and customer purchasing behavior as the dependent variable. This will help reveal more generalizable results that can be applied to many retail outlets, improving marketing performance, increasing sales, and raising profit levels.

#### ORCID

**https://orcid.org/0000-0002-8667-3219**

#### Research ethics statement

This research was conducted using an anonymous, voluntary questionnaire distributed to customers of the Al-Salam chain of stores in Kirkuk. All participants gave verbal consent, preferring not to sign any document, believing that verbal consent was sufficient and fearing that their signatures might be misused for purposes that could harm them in the future. No sensitive personal data was collected, and the confidentiality and privacy of participants’ identities were fully guaranteed.

The necessary research ethics approvals were obtained from the Ethics Approval Committee at the Center for Technical Research/Northern Technical University, under approval number (17), and a copy of this approval is attached to this document. The study was conducted in accordance with the university’s institutional research guidelines, using questionnaires as the data collection tool. However, for the purposes of transparency and verification, we have provided the ethical approval certificate obtained from the university, along with the necessary authorizations from the relevant authorities, for your review.

**The necessary research ethics approvals were obtained from the Ethics Approval Committee at the Technical Research Center/Northern Technical University, approval number (17),** and a copy of this approval is attached to this document. The study was conducted in accordance with our university’s institutional guidelines for research using questionnaires as a data collection method.

## Data Availability

All data is publicly available and cited in the research. No new data has been created, and no Digital Object Identifier (DOI) applies. The article is freely available to anyone who wishes to view and use it as a reference. Further data that researchers may require in the future can be obtained by contacting Dr. Sawsan Ibrahim Rajab, Northern Technical University, Kirkuk Polytechnic College, Kirkuk, Kirkuk Governorate, Iraq. Email: dr_sawsanibrahimrajab@ntu.edu.iq
