# The Role of Psychological Pricing Strategy in Customer Purchasing Behavior

**DOI:** 10.12688/f1000research.177273.2

**Published:** 2026-09-21

**Authors:** Asst. Prof. Dr. Sawsan Ibrahim Rajab, MOHAMMED SATWAN AMHAMMED

**Affiliations:** 1 Kirkuk Polytechnic College, Northern Technical University, Mosul, Nineveh Governorate, Iraq; 2 Hawija Technical College, Northern Technical University, Mosul, Nineveh Governorate, Iraq

**Keywords:** Keywords: Psychological pricing, fractional prices, price separators, price font size, consumer purchasing behavior, Al-Salam chain stores.

## Abstract

This study aimed to clarify the role of psychological pricing strategies, specifically fractional pricing, Decimal Point, price line size, and price line color, in guiding the customer buying behavior of Al-Salam chain stores in Kirkuk Governorate, through their combined dimensions of cognitive response, emotional response, and purchasing. The research problem stemmed from the lack of awareness among the management of Al-Salam stores in Kirkuk Governorate regarding the impact of these strategies on consumer purchasing behavior. Psychological pricing is supposed to be a tool for maximizing sales and acquiring the largest possible number of customers. Therefore, to achieve the objective and address the research problem, the descriptive-analytical approach was adopted. The study was based on two main hypotheses, which sought to determine the correlation and influence between psychological pricing strategies, represented by their dimensions (fractional pricing, price intervals, price line size, and price line color), and guiding consumer purchasing behavior.

To collect and analyze the data, a questionnaire was designed for this purpose and distributed to 400 customers of Al-Salam chain stores in Kirkuk Governorate. (340) questionnaires were returned, and among these questionnaires, (288) questionnaires were valid for statistical analysis, as all of their items were answered and no item was left unanswered. The valid questionnaire data formed the basis for reaching a set of results, which proved the existence of a strong correlation and influence of psychological pricing at the micro level, represented by (fractional prices, price interval, price line size, and price line color), and at the macro level in directing the customer’s purchasing behavior with its aggregate dimensions, represented by (cognitive response, emotional response, and purchase).

## Introduction

Marketing is a dynamic, rapidly evolving discipline continuously reshaped by structural, temporal, informational, economic, and technological paradigm shifts. In the economic arena, these transformations profoundly alter marketing theories, directly influencing consumers and their decision-making frameworks. Consequently, contemporary business research prioritizes the analysis and modulation of consumer purchasing behavior to optimize product alignment, recognizing the consumer as both the antecedent and ultimate objective considered of the marketing process.

To navigate this highly competitive landscape, enterprises implement strategic frameworks designed to acquire customers, satisfy consumer needs, and expand sales volume, profitability, and market share. Achieving these organizational objectives inherently relies on shaping consumer behavior and stimulating low-involvement, immediate purchasing decisions.

This interplay underlines the profound intersection between marketing and behavioral psychology, validating the axiom that human interaction is inherently governed by psychological principles. Accordingly, psychological pricing serves as a strategic behavioral instrument designed to evoke emotional responses and leverage cognitive biases to steer consumer purchasing decisions. Reflecting this paradigm, this study investigates the core conceptualizations of psychological pricing and its diverse strategic applications to demonstrate its efficacy in driving consumer behavior and reinforcing purchasing decisions.

## Chapter One: Research methodology

### First: Research problem

While contemporary marketing literature heavily emphasizes the paradigm shift toward consumer-centric strategies, the precise cognitive mechanisms underlying the interaction between psychological pricing and behavioral psychology remain theoretically fragmented. Extant research establishes that psychological pricing operates as a strategic behavioral tool designed to exploit consumer cognitive biases and stimulate prompt, low-involvement purchasing decisions. However, the efficacy of these strategies is not universally consistent, as consumer purchasing behavior is deeply bounded by economic volatility, informational paradigms, and distinct socio-cultural environments.

The Research Gap: A critical review of the literature reveals a significant conceptual and contextual gap. First, there are contradictory findings regarding whether psychological pricing triggers sustained brand loyalty or merely drives isolated, impulsive purchases. Second, most empirical models have been developed and validated within Western developed economies, leaving a theoretical limitation in how these behavioral heuristics manifest in emerging and transitional markets. Empirically, while localized retail entities—such as the Al-Salam chain stores in Kirkuk Governorate—intuitively adopt these tactical pricing mechanisms, there is a profound lack of empirical evidence explaining how and to what extent these psychological pricing dimensions systematically modulate or reshape consumer decision-making frameworks within this specific socio-economic context. Consequently, treating psychological pricing as a purely operational sales tool without deciphering its underlying psychological determinants creates a major disconnect between marketing theory and regional empirical reality.

The Central Research Question: Hence, this study addresses this theoretical and empirical void by investigating the following central question:

“How do alternative psychological pricing strategies systematically influence and predict consumer purchasing behavior frameworks within the distinct socio-economic context of the Iraqi retail sector, and what are the underlying behavioral mechanisms that mediate this relationship?”

Through researchers’ visits to Al-Salam chain stores in Kirkuk Governorate, it was found that although the chain’s management adopts psychological pricing, it is not fully aware of how this strategy influences consumer purchasing behavior. Psychological pricing is supposed to be their tool to achieve maximum sales and gain as many customers as possible. Hence, the study problem was identified through the following central question:

“Is there an effect of psychological pricing on consumer purchasing behavior?”

From this main question, several sub-questions arise:

1. Do customers of the Al Salam chain of stores have sufficient awareness of the role of psychological pricing in their purchasing behavior?
2. Is there an correlation and effect of fractional prices on consumer purchasing behavior?
3. Is there an correlation and effect of price separators on consumer purchasing behavior?
4. Is there an correlation and effect of price font size on consumer purchasing behavior?
5. Is there an correlation and effect of price font color on consumer purchasing behavior?

### Second: Importance of the study

The importance of this study stems from its two variables: psychological pricing is an important strategy for achieving organizational goals, while consumer purchasing behavior is one of the key aspects all organizations seek to study, analyze, and direct toward their products. In addition:

A. **Cognitive Significance**
The cognitive significance of this research stems from its attempt to enrich managerial and marketing thought through the following scientific indicators and data:
  1. **Enriching Behavioral Marketing Literature:** The research contributes to providing a comprehensive theoretical framework linking behavioral psychology with modern pricing strategies, which represents a dynamic and engaging topic in the field of consumer behavior.
  2. **Framing the Visual and Digital Dimensions of Psychological Pricing:** The theoretical significance lies in highlighting subtle details and their psychological impact on the customer—such as fractional pricing, price intervals, and the size and color of the price line—and attempting to formulate them as an integrated strategic package that influences the customer’s sensory perception.
  3. **Deconstructing the Stages of Behavioral Response:** The research contributes to bridging the knowledge gap regarding how a pricing strategy translates into a genuine customer response, by examining sequential dimensions: cognitive response, followed by emotional response, leading to the actual purchase decision.
  4. **Scarcity of Studies in the Local Environment:** This research represents a valuable scientific reference for Iraqi university libraries in general, and Kirkuk Governorate in particular, given the limited number of theoretical and empirical studies addressing the impact of visual and psychological dimensions of pricing within the retail sector and large commercial establishments in this environment.
  5. **Providing an Advanced Theoretical Measurement Model:** This research provides future researchers and graduate students with a conceptual and hypothetical model based on clear statistical indicators to measure the psychological variables governing low-involvement purchasing decisions.
B. **Practical Significance**
The practical significance of this research lies in providing empirical solutions and actionable proposals that contribute to enhancing marketing performance as follows:
  1. **Guiding Senior Management in Pricing Decisions:** This research provides a practical guide for the management of Al-Salam chain stores in Kirkuk Governorate, demonstrating how to formulate product prices using well-considered psychological and visual methods, thereby moving away from arbitrary or traditional pricing approaches.
  2. **Improving the Visual Presentation of Products:** The field study provides retail managers and merchandising supervisors with precise mechanisms to control the size and color of the price line within stores, effectively capturing customers’ attention and eliciting a positive emotional response that drives immediate purchases.
  3. **Designing Marketing Campaigns Based on Price Intervals:** The research enables management to segment and categorize its products according to price intervals and fractional pricing, making it easier for customers with varying income levels to make prompt and convenient purchasing decisions to fill their shopping carts.
  4. **Maximizing Sales and Market Share:** The applied results of the research help Al-Salam chain stores attract the largest possible number of new customers while retaining existing ones, which directly translates into increased annual profits and an enhanced competitive advantage within the markets of Kirkuk Governorate.
  5. **Providing an Empirical Measurement Tool for Evaluating Customer Behavior:** The research equips store management with a scientifically validated and pre-tested questionnaire that can be utilized periodically to assess shifts in customers’ behavioral responses (cognitive, emotional, and purchasing) and to determine their awareness of and susceptibility to the implemented marketing policies.
  6. **Raising Customer Awareness of Applied Pricing Strategies:** This involves shedding light on psychological pricing strategies and evaluating their direct impact on reshaping consumer purchasing behavior.

### Third: Research objectives

In light of the research problem and its importance, the study aims to achieve the following objectives:

1. Provide a theoretical framework defining both psychological pricing strategy and consumer purchasing behavior.
2. Identify the nature of the relationship (in terms of correlation and influence) between psychological pricing strategy (in its combined and individual dimensions) and consumer purchasing behavior.
3. Arrive at a set of findings and recommendations to improve the marketing mechanisms of business organizations in the local market, with the goal of maintaining their market share and attracting more customers.
4. Emphasize the role of applying psychological pricing strategy to increase organizations’ sales volume and achieving profits.

### Fourth: Study framework

Addressing the research problem in light of its theoretical framework and field implications requires designing a conceptual model, as shown in Figure 1, which illustrates the relationship and influence between psychological pricing strategy and consumer purchasing behavior.

**Figure 1.**
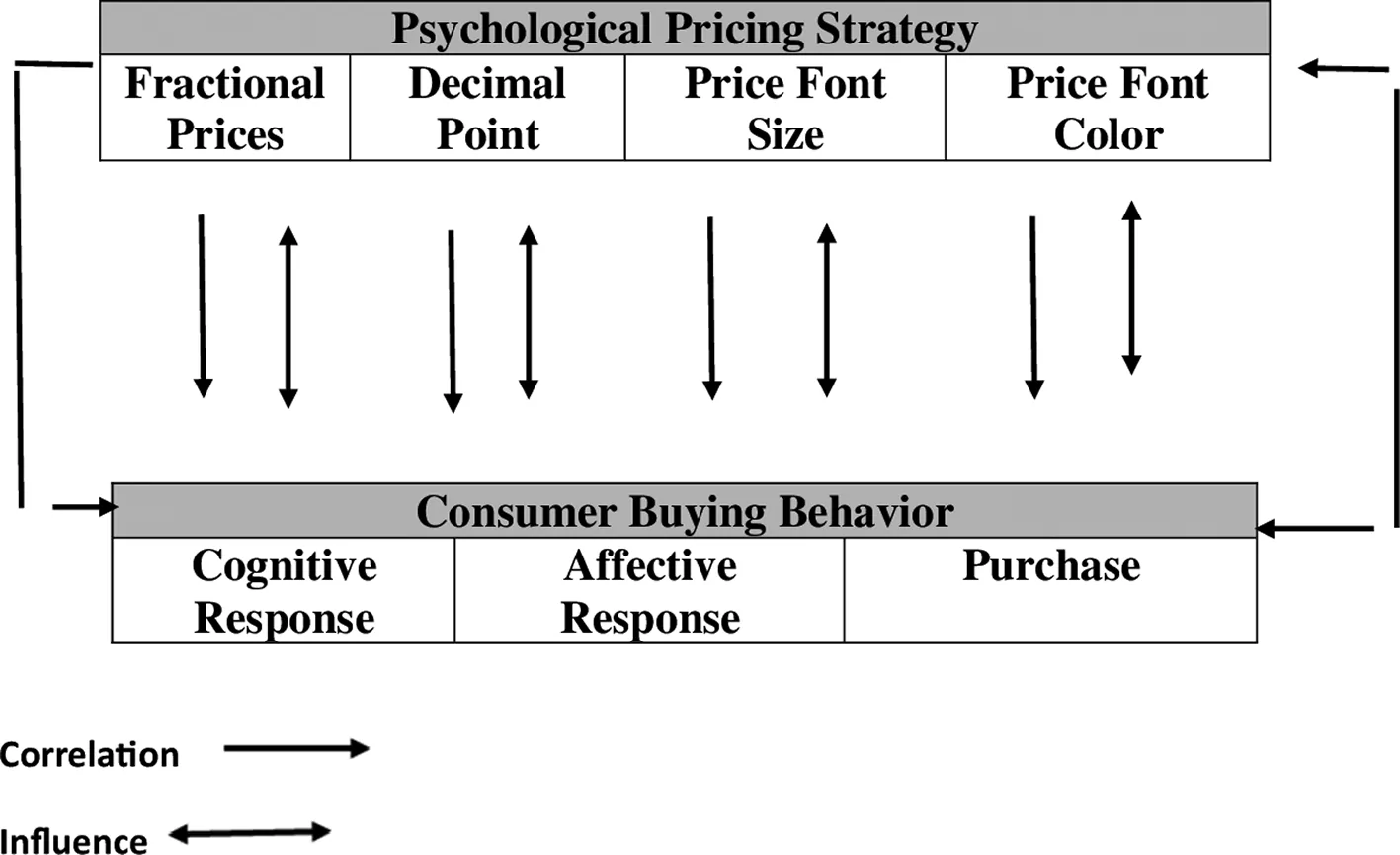
Study framework.

### Fifth: Study hypothesis

The study is based on two main hypotheses, from which sub-hypotheses branch out as follows:

### Main hypothesis one

There is a statistically significant correlation between the psychological pricing strategy (at the overall level) and customer buying behavior (at the overall level) among the customers of the researched chain. From this emerge the following sub-hypotheses:

• **Sub-hypothesis One**: There is a statistically significant correlation between the psychological pricing strategy, represented by (fractional prices), and customer buying behavior (at the overall level) among the customers of the researched chain.
• **Sub-hypothesis Two**: There is a statistically significant correlation between the psychological pricing strategy, represented by (Decimal Point), and customer buying behavior (at the overall level) among the customers of the researched chain.
• **Sub-hypothesis Three**: There is a statistically significant correlation between the psychological pricing strategy, represented by (price line size), and customer buying behavior (at the overall level) among the customers of the researched chain.
• **Sub-hypothesis Four**: There is a statistically significant correlation between the psychological pricing strategy, represented by (price line color), and customer buying behavior (at the overall level) among the customers of the researched chain.

### Main hypothesis two

There is a statistically significant positive effect of the psychological pricing strategy (at the overall level) on customer buying behavior (at the overall level) among the customers of the researched chain. From this emerge the following sub-hypotheses:

• **Sub-hypothesis One**: There is a statistically significant effect of the psychological pricing strategy, represented by (fractional price), on customer buying behavior (at the overall level) among the customers of the researched chain.
• **Sub-hypothesis Two**: There is a statistically significant effect of the psychological pricing strategy, represented by (Decimal Point), on customer buying behavior (at the overall level) among the customers of the researched chain.
• **Sub-hypothesis Three**: There is a statistically significant effect of the psychological pricing strategy, represented by (price line size), on customer buying behavior (at the overall level) among the customers of the researched chain.
• **Sub-hypothesis Four**: There is a statistically significant effect of the psychological pricing strategy, represented by (price line color), on customer buying behavior (at the overall level) among the customers of the researched chain.

### Sixth: Study methodology and data collection methods

The study adopted a descriptive-analytical approach. Its theoretical framework was built based on available relevant references, while its field component relied on a questionnaire as the primary tool for collecting and tracking data and transforming it into information. From this information, the researchers were able to deduce aspects that covered the reality of the variables adopted in the study plan. The questionnaire was designed for this purpose and used as a source for collecting data, which was then analyzed, yielding results that contributed to achieving the study’s objectives. The questionnaire was designed with clear and understandable items for the surveyed sample, and it was measurable so that the study variables could be measured in terms of structure and wording. A five-point Likert scale was used to measure the weight of the respondents’ answers. The items were (Strongly Agree, Agree, Neutral, Disagree, Strongly Disagree), with weights of (1, 2, 3, 4, 5) respectively. The tendency of respondents’ answers towards (Strongly Agree, Agree) indicates a state of agreement that reflects the availability of the variables’ dimensions, while (Neutral) indicates a state of agreement. To reflect a state of moderation, while “I disagree” and “I strongly disagree” express a state of disagreement. The responses of the chain’s customers in the research sample were analyzed using statistical tools, enabling the study to arrive at results that allowed it to offer a set of conclusions and suggestions that it hopes will be valuable to the chain’s management in its marketing efforts.

### Seventh: Study boundaries

• **Time Boundaries**: The study aims to explore customers’ opinions during the specified period from 2/1/2025 to 4/1/2025.
• **Spatial Boundaries**: The study was limited to the branches of Al-Salam chain stores inside Kirkuk Governorate, totaling (4) branches, which served as the field of the study.
• **Human Boundaries**: A total of (400) questionnaires were distributed to members of the Al Salam chain stores who expressed their consent and willingness to cooperate and answer the questionnaire, and (340) questionnaires were returned. The questionnaires that were valid for statistical analysis and which the respondents answered in full were (288) questionnaires.

### Eighth: Description of the study field, population, and sample

**A: Introductory Overview of Al-Salam Retail Chain in Kirkuk Governorate**

Al-Salam chain stores was established in Kirkuk Governorate in (2003). It has (10) branches, (4) of which are inside Kirkuk city, while the rest are distributed across the main districts of the governorate. These branches offer a wide variety of branded products from different origins, all of which relate to the essential needs of Iraqi families. A large number of customers frequent these stores due to the availability of diverse product lines that meet their needs and satisfy their desires.

All stores are managed by a Board of Directors that focuses on developing business plans and making the necessary decisions for the chain’s growth, sustainability, and profitability, thereby ensuring its continuity and strengthening its competitive position in the local Kirkuk market compared to other retail chains operating in the same governorate.

The stores employ a sufficient number of experienced and qualified sales staff who provide various services to facilitate customer access to products, in addition to offering necessary information about any displayed product when needed.

**B: Description of the Study Population and Sample**

The study population was defined as the customers shopping at the (4) branches of Al-Salam chain stores in central Kirkuk. For the purpose of distributing the questionnaire to shoppers, four employees were appointed and distributed across the four branches. Their task was to receive customers, explain the purpose of the questionnaire, and encourage them to participate by answering its items. However, they faced difficulty in convincing many shoppers because the majority of them did not want to participate by expressing their opinions, or because they did not understand their role in building future pricing strategies. Some made excuses by saying that they did not have enough time to participate. Only (400) questionnaires were distributed to a diverse sample questionnaires were distributed among a diverse sample of customers shopping for different products of various brands and origins displayed by the chain, those who agreed to participate by stating their opinions on the questionnaire items. (288) valid questionnaires were retrieved for statistical analysis. Table 1 shows the demographic data of the sample studied.

**Table 1.** Demographic data for the sample group.

#	Demographic data	Categories	Number	%
1	Gender	Female	167	57
		Mal	121	42
2	Age group	(18-28) years	43	15
		(29–39) years	92	32
		(40–50) years	97	34
		(51) years and over	56	19
3	Social status	Married	193	67
		Single	95	33

Source: Researchers’ calculations based on AMOS V24 results N = 288.

## Chapter One: Theoretical framework

### Section One: Psychological pricing

**First: Psychological Pricing – Concept, Importance, and Objectives**

Psychological pricing is considered an important concept in marketing and business management, playing a crucial role in determining product prices. Psychological pricing refers to a “policy that relies primarily on price to influence the psychological state of the customer, where the price is adjusted according to customer type. For some, high prices mean something, while others may be attracted to anything advertised at a low price”. ^1^ It is also defined as “the process of setting prices based on customer lists, where it depends on their emotional responses, subjective evaluations, and feelings toward specific purchases”. ^2^ According to ^3^ it is “policies that rely on influencing the customer’s psychological state to drive them to make a purchase decision as a result of an emotional reaction rather than depending on rational thought in purchasing.”

Accordingly, psychological pricing can be defined as “a pricing strategy that uses specific methods to influence customer perceptions and behavior, exploiting and employing their emotional responses to certain price points, making products appear more attractive, thereby generating a desire to purchase.”

Businesses adopt psychological pricing within their marketing offers due to its importance, which can be clarified as follows ^4^:

1. Enables the organization to identify what customers like in its products. During marketing campaigns, they focus on appealing to customer emotions by knowing their preferences and emphasizing what benefits them.
2. Maintain continuous communication with customers to build closeness.
3. Use multiple methods in product marketing based on marketing mix elements, all adjusted to psychological influences.
4. Attract customers by focusing on the emotional and psychological side.
5. Present marketing offers to customers in an attractive way.
6. Study the psychological impact of surrounding environmental influences on customers.
7. Identify what might prevent customers from making purchasing decisions, which affects marketing activities and operations.
8. Help improve marketing campaigns and strategies for more effective influence on both customers and the purchasing process.
9. Study customer psychology, decision-making, and preferences among various brands.

The use of psychological pricing as part of promotional tools helps organizations achieve several goals, including ^5^:

1. Simplifying the purchase decision-making process for the customer and identifying cost-conscious customers, as this is one of their main weaknesses. With the displayed price, they spend less time thinking, enabling them to make a purchase decision quickly as an opportunity to seize.
2. Designing discount systems with distinctive impact, influencing the customer’s emotional desire to purchase.
3. Making the customer feel intelligent and successful, as though they got a good deal and outsmarted the general pricing system followed by competing organizations offering similar products at higher prices. ^6^
4. Helping the organization determine the cost of products it intends to sell, thereby enabling it to set a more effective marketing strategy for its products.
5. Enhancing sales, raising levels, and enabling a wider segment of customers to purchase these products.
6. Building a good reputation and a positive brand image in the customer’s mind, positioning the organization as one that wants everyone to have access to good products at reasonable prices that meet desires, satisfy needs, and make the customer feel they achieved the best purchase. ^7^

**Second: Psychological Pricing Strategies**

There are multiple psychological pricing strategies, and organizations choose the strategy that best suits their internal and external conditions. They may also reconsider any psychological pricing strategy currently being followed, based on changes and updates in the internal and external marketing environment, and adopt another strategy more aligned with these changes ^10, 8 9^ identified psychological pricing strategies as (Fractional Prices, Comma Effect, Font Size Effect, Price Anchoring), ^11, 12^ and agree with him in identifying the same strategies that can be adopted within psychological pricing, while ^13^ identified psychological pricing strategies as (Fractional Prices, Price Interval, Hundred Rule for Discounts, Word Free, Font Size When Writing Old and New Price, Red Color and its Effect on Writing Price, Effect of Descending Order of Prices).

Based on the above, we see that there is agreement on identifying some psychological pricing strategies that the organization can adopt in its marketing program, certainly in accordance with the requirements and challenges of its internal and external environment. For the purposes of this research, we will address the strategies of (fractional pricing, price interval, price line size, and price line color), as follows ^14^:

**1- Fractional Pricing**

According to this strategy, prices are called “attractive prices.” Through this strategy, the organization attempts to influence the customer psychologically by suggesting that the product is lower in price because it is slightly less than a round number. ^14^ The organization uses fractions of the currency unit, such as stating that the selling price of a unit is $3.99 instead of $4. This fractional number gives the customer the impression that prices have been reduced and set with great precision. ^15^

**2- Decimal Point**

According to this strategy, the organization believes that if it places a punctuation mark, the customer will perceive the price differently. For example, when a product is priced as (1500$), the customer sees it differently compared to (1.500$). Although any rational person knows they are the same amount, this small punctuation mark creates a strong difference in the customer’s perception of the price. Most customers perceive prices written with punctuation as higher than those without, as people interpret numbers visually, which influences their purchase decisions. ^16^

**3- Price Font Size**

Reducing the font size in which the price is written on the package directly, or on the tag placed on the product, is one of the most effective strategies for increasing sales. Products with smaller-sized price fonts are much more likely to be purchased than those with large-sized price fonts. This strategy is based on the idea that smaller font sizes are psychologically associated with lower prices. In the customer’s mind, font size is linked with value—smaller font suggests a smaller price, and vice versa. ^17^

**4- Price Font Color**

The color of the price font significantly affects customer behavior and purchase decisions. The colors used to write prices on displayed products can trigger different feelings and emotions, influencing how customers interact with prices and whether they will purchase the product. Red, for example, is often used by marketers on discount tags to grab attention and stimulate the desire to buy, while blue may be used for luxury products to suggest quality and trust. ^18^

## Axis Two: Customer buying behavior

### First: The concept and importance of customer buying behavior

Understanding customer behavior is a crucial stage in the marketing process. This process, driven by the need to meet customer demands, is by no means easy. Customers, for example, may express interest in buying a product but not actually purchase it, ^19^ or they may not know exactly what they want to buy. They may also make their decisions at the last minute depending on their circumstances. Therefore, marketing management must analyze customer needs and understand how they perceive things—this means paying attention to their buying behavior. ^20^

**Customer buying behavior has been defined as:**

• “The set of actions a customer undertakes when choosing a product available in the market—the feelings and thoughts the customer relies on before or during the purchase of any product. The final outcome of this behavior is the customer’s ultimate decision regarding product selection, brand preference, choice of sales outlet, timing of purchase, and frequency of purchase”. ^21^
• “The mental processes and behavioral actions the customer undertakes when intending to buy a product, as they search for a product that achieves the desired level of satisfaction for their needs and desires and aligns with their expectations”. ^22^
• ^23^defines it as “the behaviors or stages an individual goes through when making a purchase decision for a product in order to satisfy their needs. Brand owners study customer behavior to design strategies and advertising campaigns directed at customers”.

The importance of studying customer buying behavior lies in the fact that it achieves several objectives for both the customer and the organization, as follows ^24^:

1. Helps the organization design products in ways that ensure customer acceptance, thereby increasing acquisition rates. The more the organization understands its customers, the better it can serve and satisfy their needs and achieve its goals.
2. Enables the organization to discover new marketing opportunities by researching unmet and emerging customer needs and investing in them to diversify its products, enhance competitiveness, and increase market share.
3. Understanding customer behavior and their purchasing power helps the organization set pricing policies. A successful organization is one that offers products that satisfy customer desires within their purchasing capacity.
4. Enables the organization to evaluate its marketing performance and identify strengths and weaknesses by knowing customers’ opinions about its products.
5. Helps determine distribution channels for its products—whether by relying on its own outlets with a focus on personal selling and sales representatives for direct customer interaction, or by relying on intermediaries and agents (wholesalers and retailers) for indirect interaction. ^25^
6. Assists the organization in designing promotional policies. By understanding customer tastes and preferences, marketing management can determine a suitable promotional mix aimed at influencing and persuading customers to buy its products. ^26^

### Second: Dimensions of customer buying behavior

Customer purchasing behavior can be measured through several dimensions, and both ^20^^,^^24^^,^^27^ agreed upon a set of dimensions, which will also be adopted in this study due to their consistency with the data. These dimensions include the following:

• **Cognitive Response**: The starting point of any customer behavior is an unfulfilled need. Marketing programs show the importance of a product, as well as its features and benefits. Once the customer recognizes the need for a product, they begin to gather information about it to determine if it meets their need. ^28^ Customers rely on multiple sources to support what they already know, such as their social environment, which provides information that organizations should use in marketing programs to differentiate their products from competitors, giving customers knowledge that makes them prefer the organization’s product. ^29^
• **Affective Response**: Emotional response reflects the customer’s feelings and emotions toward the purchase subject. If the customer feels liking or disliking, they may develop positive or negative attitudes toward the product. The emotional relationship the customer forms with the product is complex, involving multiple behavioral levels and a mix of conscious and unconscious internal and external reactions. Product evaluation is influenced by logical judgments (e.g., performance, ease of use, necessity of ownership) as well as subjective ones (e.g., lack of rational justification but strong personal desire to own it). ^30^
• **Purchase**: The buying decision process is defined as the procedure through which customers collect and analyze information and choose among product or organizational alternatives. It also consists of coordinated measures taken to satisfy needs. ^31^ Many situational factors affect the purchase decision, such as time pressure. For example, limited time may lead to a hasty decision without sufficient information to evaluate alternatives, possibly resulting in customer dissatisfaction post-purchase. ^32^

### Section Three: Field framework

In this section, the study hypotheses will be tested by determining and analyzing the correlation and impact relationship between psychological pricing strategies and customer buying behavior, as follows:

**First: The Relationship Between Psychological Pricing Strategies and Customer Buying Behavior**

• **Main Hypothesis 1**: There is a statistically significant correlation between psychological pricing strategies (at the overall level) and customer buying behavior (at the overall level) among the customers of the studied chain
• The results of Table 2 show the existence of a positive, statistically significant correlation between the independent variable (psychological pricing strategy) and the dependent variable (customer purchasing behavior), as indicated by the overall correlation coefficient of (0.91). This reflects a strong positive relationship between the study’s variables, based on the probability value (P-value), which was found to be (0.002), less than (0.05). Moreover, the similarity of the signs of both the Upper and Lower limits of the 95% Confidence Interval at the significance level (0.005) further supports this This relationship points to psychological pricing as one of the important strategies that enables the research chain to offer its products at attractive prices that encourage customers to buy. Accordingly, the first main hypothesis is accepted, which states:
• “There is a statistically significant correlation between the psychological pricing strategy (at the overall level) and customer buying behavior (at the overall level) among the customers of the studied chain.”

**Table 2.** Correlation between psychological pricing strategy and consumer buying behavior.

First variable	Direction of relationship	Second variable	Correlation value	95% Confidence interval		P - value
				Upper	Lower	
Psychological Pricing Strategy	→←	Consumer Buying Behavior	0.91	0.951	0.845	0.002

Source: Researchers’ calculations based on AMOS V24 results (α ≤ 0.05, n = 288).

**2- The sub-hypotheses derived from the first main hypothesis**

Based on the data in Table 3, the following becomes clear:

• **First Sub-Hypothesis **:
• There is a statistically significant positive correlation between the psychological pricing strategy, represented by fractional prices, and customer buying behavior (at the overall level) among the customers of the studied chain.
• A statistically significant positive relationship exists between fractional prices and purchasing behavior, as indicated by the correlation coefficient of (0.91). This relationship is significant based on the P-value, which was (0.003), less than (0.05). Moreover, the signs of both the Upper and Lower limits of the (95% Confidence Interval) at the significance level (0.05) were similar. This relationship suggests that adopting fractional prices motivates customers and directs their purchasing behavior, as they perceive it as an opportunity to achieve savings when buying. Accordingly, the first sub-hypothesis derived from the first main hypothesis is accepted.
• **Second Sub-Hypothesis:**
• There is a statistically significant correlation between the psychological pricing strategy, represented by Decimal Point, and customer buying behavior (at the overall level) among the customers of the studied chain.
• A statistically significant positive relationship exists between Decimal Point and purchasing behavior, as indicated by the correlation coefficient of (0.79). This relationship is significant based on the P-value, which was (0.002), less than (0.05). Moreover, the signs of both the Upper and Lower limits of the (95% Confidence Interval) at the significance level (0.05) were similar. This relationship shows that Decimal Point play a role in guiding customer buying behavior, encouraging them to buy more products. Therefore, the second sub-hypothesis derived from the first main hypothesis is accepted.
• **Third Sub-Hypothesis:**
• There is a statistically significant correlation between the psychological pricing strategy, represented by price font size, and customer buying behavior (at the overall level) among the customers of the studied chain.
• A statistically significant positive relationship exists between price font size and customer buying behavior, as indicated by the correlation coefficient of (0.82). This relationship is significant based on the P-value, which was (0.003), less than (0.05). Moreover, the signs of both the Upper and Lower limits of the (95% Confidence Interval) at the significance level (0.05) were similar. This result indicates that the size of the font in which the price is written on displayed products plays a role in guiding customer behavior toward buying, as smaller font sizes are perceived to be associated with lower prices. Based on this, the third sub-hypothesis derived from the first main hypothesis is accepted.
• **Fourth Sub-Hypothesis:**
• There is a statistically significant correlation between the psychological pricing strategy, represented by price font color, and customer buying behavior (at the overall level) among the customers of the studied chain.
• A statistically significant positive relationship exists between price font color and customer buying behavior, as indicated by the correlation coefficient of (0.68). This relationship is significant based on the P-value, which was (0.002), less than (0.05). Moreover, the signs of both the Upper and Lower limits of the (95% Confidence Interval) at the significance level (0.05) were similar. This result shows that using colors in writing prices, or highlighting a specific feature of the buying, captures the customer’s attention and encourages them to purchase. Accordingly, the fourth sub-hypothesis derived from the first main hypothesis is accepted.

**Second: The Effect of Psychological Pricing Strategy on Customer Purchasing Behavior**

**Table 3.** Correlation between dimensions of psychological pricing strategy and consumer buying behavior.

First variable	Direction of relationship	Second variable	Correlation value	95% Confidence interval		P – value
				Upper	Lower	
Psychological Pricing Strategy	→←	Consumer Buying Behavior	0.91	0.956	0.843	0.003
Fractional Prices	→←		0.79	0.884	0.683	0.002
Decimal Prices	→←		0.82	0.887	0.720	0.003
Price Font Size	→←		0.68	0.791	0.555	0.002
Price Font Color	→←		0.91	0.956	0.843	0.003

Source: Researchers’ calculations based on AMOS V24 results AMOS V24 (α ≤ 0.05, n = 288).

**1- Second Main Hypothesis:**

There is a statistically significant positive effect of the psychological pricing strategy (at the overall level) on customer buying behavior (at the overall level) among the customers of the studied chain.

Table 4: The Effect of Psychological Pricing Strategy on Customer Purchasing Behavior.

**Table 4.** Effect of psychological pricing strategy on consumer buying behavior.

Independent variable	Direction of effect	Dependent variable	Regression coefficient estimate (β)	Standard error of β (Se.(β))	R ^2^ (Coefficient of determination)	Critical ratio (C.R.)	P - value
Psychological Pricing Strategy	-->	Consumer Buying Behavior	0.907	0.028	0.82	32.392	0.002

Critical t-value: (t Tab = 1.96).

Source: Researchers’ calculations based on AMOS V24 results (α ≤ 0.05, n = 288).

The results shown in Table 4 indicate the existence of a substantial effect of the independent variable, “psychological pricing strategy” (at the overall level), on the dependent variable, “customer buying behavior” (at the overall level). This is evidenced by the value of (β), which reached (0.907). This effect is statistically significant as indicated by the P-value of (0.002), which is less than (0.05). The same result is also confirmed by the (C.R.) value, which reached (32.392), exceeding the tabulated t-value (t Tab) of (1.96).

Furthermore, the value of (R ^2^), which amounted to (0.82), indicates a significant effect of the independent variable on the dependent variable. This means that the independent variable (psychological pricing strategy) explains (82%) of the variance in the dependent variable (customer buying behavior), while the remaining percentage is attributed to factors beyond the scope of this study. This result shows that customer buying behavior is clearly influenced by psychological pricing strategies, thus motivating them to buy. Accordingly, the second main hypothesis is accepted.

**2- The sub-hypotheses derived from the second main hypothesis**

Based on the data presented in Table 5, the following becomes clear:

**Table 5.** The existence of the dimensions of the psychological pricing strategy in customer purchasing behavior.

Independent variable	Direction of effect	Dependent variable	Regression coefficient estimate (β)	Standardized Regression Weight (SRW)	Standard error of β (Se.(β))	Coefficient of determination (R ^2^)	Critical ratio (C.R.)	P-value
Fractional Prices	-->	Consumer Buying Behavior	0.909	1.091	0.135	0.87	3.117	0.003
Decimal Prices	-->		0.797	0.831	0.106		2.920	0.002
Price Font Size	-->		0.685	0.601	0.079		7.607	0.002
Price Font Color	-->		0.824	0.829	0.092		9.010	0.003

Source: Researcher’s calculations based on AMOS V24 results (α ≤ 0.05, n = 288) Critical t-value: t Tab = 1.96.

**• First Sub-Hypothesis:**

There is a statistically significant effect between the psychological pricing strategy, represented by (fractional pricing), and customer buying behavior (at the aggregate level) among the customers of the studied chain.

There is an effect of the fractional pricing dimension on customer buying behavior, as indicated by the value of (β), which reached (1.091). This effect is statistically significant, with a p-value of (0.003), which is less than (0.05). The same result is confirmed by the C.R. value, which reached (3.117), greater than the tabulated t-value (1.96), These results show that customers expect to achieve greater savings when buying products priced using fractional pricing. This leads us to accept the first sub-hypothesis derived from the second main hypothesis.

**• Second Sub-Hypothesis:**

There is a statistically significant effect between the psychological pricing strategy, represented by (Decimal Point), and customer buying behavior (at the aggregate level) among the customers of the studied chain.

There is an effect of Decimal Point dimension on customer buying behavior, as indicated by the value of (β), which reached (0.831). This effect is statistically significant, with a p-value of (0.002), which is less than (0.05). The same result is confirmed by the C.R. value, which reached (2.920), greater than the tabulated t-value (1.96), These results show that the customer feels that the prices determined by using the Decimal Point the true price of the product and that their purchase of this product aligns with their personal capabilities and desires. This leads us to accept the second sub-hypothesis derived from the second main hypothesis.

**• Third Sub-Hypothesis:**

There is a statistically significant effect between the psychological pricing strategy, represented by (price font size), and customer buying behavior (at the aggregate level) among the customers of the studied chain.

There is an effect of the price font size dimension on customer buying behavior, as indicated by the value of (β), which reached (0.601). This effect is statistically significant, with a p-value of (0.002), which is less than (0.05). The same result is confirmed by the C.R. value, which reached (7.607), greater than the tabulated t-value (1.96), These results show that the font size used in product pricing attracts attention. When the new price of a product is displayed in a font size different from the size used for the old price, it ensures that the customer will notice the price, and this can stimulate their desire to buy due to the difference between the old and new prices Thus, the customer has made a good deal when purchasing the product. This leads us to accept the third sub-hypothesis derived from the second main hypothesis.

**• Fourth Sub-Hypothesis:**

There is a statistically significant effect between the psychological pricing strategy, represented by (price font color), and customer buying behavior (at the aggregate level) among the customers of the studied chain.

There is an effect of the price font color dimension on customer buying behavior, as indicated by the value of (β), which reached (0.829). This effect is statistically significant, with a p-value of (0.003), which is less than (0.05). The same result is confirmed by the C.R. value, which reached (9.010), greater than the tabulated t-value (1.96), These results show that colors have clear effects on customer decisions. Adopting a different color for the new price than the old price color draws the customer’s attention to the fact that there are price reductions and the purchase process will be profitable for him. Also, adopting a specific color for pricing some products, such as luxury or distinctive products, makes the customer feel the value of these products and motivates him to buy. This leads us to accept the fourth sub-hypothesis derived from the second main hypothesis.

## Chapter Four: Conclusions and recommendations

### First: Conclusions

1. Customer buying behavior in its dimensions is of great interest to the management of Al-Salam chain stores in Kirkuk, as the customer is the foundation for the chain’s survival, growth, and continuity. Moreover, the customer is the focus of all the marketing activities adopted by the chain, and all its efforts are directed at satisfying the customer and persuading them to purchase.
2. Psychological pricing is one of the important strategies that enables organizations in general to offer their products at attractive prices that encourage customers to buy.
3. There is a significant positive correlation between psychological pricing (with its dimensions: fractional pricing, price separator, price font size, and price font color) and customer buying behavior (with its dimensions: cognitive response, **Affective Response**, and purchasing) in the studied chain.
4. There is a significant positive correlation between each individual dimension of psychological pricing (fractional pricing, **Decimal Point**, price font size, and price font color) and customer buying behavior (cognitive response, **Affective Response**, and purchasing) in the series under study. The fractional prices dimension had the highest correlation, which shows that its adoption motivates customers and directs their behavior towards purchasing as an opportunity through which savings are achieved when purchasing. The line color dimension had the least correlation, despite its role in directing the customer’s purchasing behavior.
5. There is a significant positive effect of psychological pricing, across both its and combined dimensions (fractional pricing, **Decimal Point**, price font size, and price font color), on customer buying behavior across its aggregate dimensions (cognitive response, **Affective Response**, and purchasing) within the stores under study. This demonstrates that customer buying behavior can be effectively directed through the strategic application of these psychological pricing dimensions.
6. Fractional pricing ranked first in terms of its impact on customer purchasing behavior, driven by its inherent psychological appeals that stimulate the customer’s desire to buy. Conversely, price font size emerged as the least influential factor in shaping customer buying behavior, notwithstanding its role in capturing customer attention to price differentials and motivating conversion.
7. Utilizing fractional numbers in displaying product prices leads customers to believe that the pricing process is precise and objective.
8. The use of a Decimal Point while displaying the subsequent digits in a distinct font size and color is more impactful and capturing than presenting a whole price without decimals for the same product, thereby enhancing customer value perception and making the items appear more affordable.
9. Writing the price in a clear, large, or different font style draws customers’ attention to products and generates in them the desire to make a purchase.

### Second: Recommendations

In light of the study results and conclusions, it is appropriate to present the following recommendations:

1. The management of Al-Salam chain stores should pay greater attention to psychological pricing, as it is one of the most important pricing strategies that primarily address the psychological side of the customer, driving them to make a purchase decision based more on emotional and psychological reaction than logical reasoning.
2. The management of the studied chain stores should recognize that psychological pricing strategy elevates the value of the product in customers’ perception by instilling positive feelings when they see the products and their prices; since customers purchase more with emotions than through deep rational thought.
3. Using psychological pricing as a pricing strategy by Al-Salam chain stores management has a significant impact on increasing sales, and consequently increasing both profits and market share.
4. The management of Al-Salam chain stores should give greater attention to psychology and its influence on marketing in general and pricing in particular, as it is a science based on marketing strategies that rely on purely psychological studies of the customer. This enables the chain to attract more customers and influence their buying behavior.
5. The chain’s management should pay more attention to the dimensions of psychological pricing strategy to enhance its role in achieving better results regarding guiding customer buying behavior.
6. The chain’s management should be encouraged to focus on broader dimensions of customer buying behavior, studying and employing them as a competitive tool against rival stores.
7. The management of Al-Salam chain stores should seek assistance from psychology experts, as this will bring them closer to the customer and make it easier to understand their needs and desires, thereby providing them with the best offerings. This should be based on psychological research tailored to the local customer environment in order to achieve the best possible results.
8. The studied chain’s management should conduct ongoing study of the relationship between the dimensions of psychological pricing strategy and customer buying behavior.
9. Employees of Al-Salam chain stores should be trained in customer interaction techniques and how to motivate and encourage customers to purchase.

### Limitations and future research

Although the study contributed to achieving its stated objectives, there are several limitations that must be acknowledged. These limitations are as follows:

1. Data was collected from customers of the studied retail chain. Although the number of participants reached 400, this number is insufficient given the large customer base of this chain. The refusal to participate stemmed from several reasons, including apprehension about participating, time constraints, and a lack of awareness of the importance of scientific research and its role in improving marketing performance. Therefore, generalizing the results is certainly appropriate for the studied retail chain, but it is essential to expand the study to include other retail centers if they wish to utilize the findings in their marketing operations and activities.

2. Verbal consent was obtained from customers who participated in answering the questionnaire items. This was due to their reluctance to write their names and sign any other document, whether it be the questionnaire or otherwise, with their name and legal information, fearing its potential misuse for purposes that could harm them in the future. Therefore, verbal consent was sufficient to ensure cooperation and completion of the questionnaire.

3. The study relied on self-reported measures, which may introduce bias into the responses or influence social desires that could affect the accuracy of participants’ answers.

4. The research focused primarily on psychological pricing strategies and their impact on customer purchasing behavior. It did not address other pricing strategies or factors that may influence customer purchasing behavior.

5. Although many studies have examined customer buying behavior, several new factors have emerged as a result of technological advancements and modern communication methods. These factors should be added to those mentioned previously in this study, and further research is needed.

6. Research should aim to study and analyze the causal relationships that can influence customer buying behavior through the application of more comprehensive psychological pricing strategies across diverse or specific customer groups and in different social environments. This will help identify causal pathways and clarify the nature of the correlation and influence between them.

7. Studies should employ a mediating or moderating variable between psychological pricing strategies as the independent variable and customer buying behavior as the dependent variable. This will help reveal more generalizable results that can be applied to many retail outlets, improving marketing performance, increasing sales, and raising profit levels.

#### ORCID

**https://orcid.org/0000-0002-8667-3219**

#### Research ethics statement

This research was conducted using an anonymous, voluntary questionnaire distributed to customers of the Al-Salam chain stores in Kirkuk. All participants gave verbal consent, preferring not to sign any document, believing that verbal consent was sufficient and fearing that their signatures might be misused for purposes that could harm them in the future. No sensitive personal data was collected, and the confidentiality and privacy of participants’ identities were fully guaranteed.

The necessary research ethics approvals were obtained from the Ethics Approval Committee at the Center for Technical Research/Northern Technical University, under approval number (17), and a copy of this approval is attached to this document. The study was conducted in accordance with the university’s institutional research guidelines, using questionnaires as the data collection tool. However, for the purposes of transparency and verification, we have provided the ethical approval certificate obtained from the university, along with the necessary authorizations from the relevant authorities, for your review.

## Data Availability

All data is publicly available and cited in the research. No new data has been created, and no Digital Object Identifier (DOI) applies. The article is freely available to anyone who wishes to view and use it as a reference. Further data that researchers may require in the future can be obtained by contacting Dr. Sawsan Ibrahim Rajab, Northern Technical University, Kirkuk Polytechnic College, Kirkuk, Kirkuk Governorate, Iraq. Email: dr_sawsanibrahimrajab@ntu.edu.iq
