# Supplementary material for: The Role of Psychological Pricing Strategy in Customer Purchasing Behavior
Source: F1000Res. 2026 Oct 6;15:855. Originally published 2026 Jun 1. [Version 3] doi: 10.12688/f1000research.177273.3 (PMC13627935; doi:10.12688/f1000research.177273.3)
Supplement: Peer review history

# Reviewer report: Reviewer response for version 2

**DOI:** 10.5256/f1000research.210210.r523275

**Published:** 2026-09-30

**Authors:** Munther Khuder Yaqoob

**Affiliations:** 1 Marketing Management, University of Mosul, Mosul, Nineveh Governorate, Iraq

Hi Sir, 

 The revised research paper has been reviewed and found to be appropriate given the feedback received. The researchers have made adjustments that may improve the quality of the research according to scientific methodology.

 thankyou

Is the work clearly and accurately presented and does it cite the current literature?

Yes

If applicable, is the statistical analysis and its interpretation appropriate?

Yes

Are all the source data underlying the results available to ensure full reproducibility?

Yes

Is the study design appropriate and is the work technically sound?

Yes

Are the conclusions drawn adequately supported by the results?

Partly

Are sufficient details of methods and analysis provided to allow replication by others?

Yes

Reviewer Expertise:

Marketing Management, Digital Marketing, E- Marketing, Customer Behavior, and Marketing Strategies and Brand.

I confirm that I have read this submission and believe that I have an appropriate level of expertise to confirm that it is of an acceptable scientific standard.

# Reviewer report: Reviewer response for version 2

**DOI:** 10.5256/f1000research.210210.r523274

**Published:** 2026-09-29

**Authors:** Ahmed Hammadi

**Affiliations:** 1 University of Fallujah, Fallujah, Iraq

I have reviewed the revised article and the authors’ responses to the previous review comments. The authors have adequately addressed my previous comments, and I have no further comments to make on the revised manuscript.

Is the work clearly and accurately presented and does it cite the current literature?

Yes

If applicable, is the statistical analysis and its interpretation appropriate?

Partly

Are all the source data underlying the results available to ensure full reproducibility?

Partly

Is the study design appropriate and is the work technically sound?

Yes

Are the conclusions drawn adequately supported by the results?

Partly

Are sufficient details of methods and analysis provided to allow replication by others?

Partly

Reviewer Expertise:

Marketing management, digital marketing, consumer behavior, strategic management, and management information technology.

I confirm that I have read this submission and believe that I have an appropriate level of expertise to confirm that it is of an acceptable scientific standard.

# Reviewer report: Reviewer response for version 2

**DOI:** 10.5256/f1000research.210210.r523273

**Published:** 2026-09-22

**Authors:** George Spais

**Affiliations:** 1 Hellenic Open University, Patras, Greece

Thank you for revising the manuscript. However, your response does not provide a point-by-point explanation of the revisions or indicate where you made specific changes. Although you now acknowledge the self-report limitations and report ethics approval, my principal concerns remain substantially unresolved.

 I find the prose remains repetitive and grammatically weak, terminology remains inconsistent, and causal language persists despite the cross-sectional survey design. Methodological and statistical reporting also remains inadequate. You do not provide convincing evidence of measurement reliability, construct validity, sampling adequacy, or model diagnostics. Table-text inconsistencies persist, including incorrect table references, shifting construct labels, and the questionable coefficient β = 1.091.

 I also find the theoretical framework largely descriptive, with insufficient engagement with contemporary international research. Most importantly, the data availability statement still claims that “no new data has been created,” although the authors collected original questionnaire data, while the underlying data remain unavailable. Reference relevance and accuracy also require substantial correction.

 Consequently, I conclude that the revision does not adequately address my original concerns, and my recommendation remains Not Approved.

Is the work clearly and accurately presented and does it cite the current literature?

Partly

If applicable, is the statistical analysis and its interpretation appropriate?

Partly

Are all the source data underlying the results available to ensure full reproducibility?

No

Is the study design appropriate and is the work technically sound?

Partly

Are the conclusions drawn adequately supported by the results?

Partly

Are sufficient details of methods and analysis provided to allow replication by others?

No

Reviewer Expertise:

promotion management, digital, promotions, digital marketing and marketing educations

I confirm that I have read this submission and believe that I have an appropriate level of expertise to state that I do not consider it to be of an acceptable scientific standard, for reasons outlined above.

# Response

**Published:** 2026-09-22

**Authors:** Sawsan Ibrahim Rajab

**Affiliations:** Marketing management technique, Northern Technical University / Kirkuk Polytechnic College, Kirkuk, Kirkuk, Iraq

**Dear Dr. George Spais,**

 We would like to express our deepest gratitude and sincere appreciation for the exceptional time, effort, and thorough academic care you invested in reviewing the second version of our manuscript. Your highly constructive, precise, and rigorous feedback has been invaluable in elevating the scientific quality, methodological clarity, and empirical accuracy of our research.

 We have taken all your principal concerns with the utmost seriousness. In this third version (Version 3), we have executed substantial revisions to address every single comment point-by-point, including rectifying statistical transpositions, completely updating the theoretical framework with genuine, foundational, and contemporary literature (2024–2026), and clarifying our data protocols based on local field constraints.

 We sincerely hope that these rigorous improvements meet your high academic standards and demonstrate our absolute commitment to scientific excellence. Thank you once again for your invaluable guidance, which has tremendously reshaped and strengthened this paper.

 Best respectful regards,

**Dr. Sawsan Ibrahim Rajab**

**(Corresponding Author)**

**Northern Technical University, Kirkuk Polytechnic College, Iraq.**

**Point-by-Point Response Table**

**1. Evaluation Status & The Point-by-Point Requirement**

- **Reviewer’s Concern:** The reviewer noted that the previous response did not provide a clear, point-by-point explanation of the revisions or indicate where specific changes were made.
- **Author’s Response:** We sincerely apologize for this previous omission. We have now structured this comprehensive point-by-point response document to transparently track and detail every single adjustment made, cross-referenced with exact manuscript locations.

**2. The Statistical Value (β = 1.091) and Table Inconsistencies**

- **Reviewer’s Concern:** The reviewer questioned the statistically impossible coefficient β = 1.091 in Table 5 and pointed out shifting construct labels.
- **Author’s Response:** We are deeply grateful for your sharp eye in spotting this critical error. There was an inadvertent column transposition during the layout drafting of Table 5. Mathematically, **1.091** is the **Unstandardized Regression Coefficient (B)** (which naturally yields the correct Critical Ratio reported: 1.091 ÷ 0.135 = 3.117). Conversely, the actual **Standardized Regression Weight (SRW / β)** for Fractional Prices is **0.909**, which is fully valid and compliant (< 1.0).
- **Revision Made:** In Version 3, Table 5 and its accompanying explanatory text have been thoroughly corrected by swapping the transposed values into their proper columns. All construct labels, numerical figures, and mathematical references have been strictly synchronized across the paper.

**3. Causal Language vs. Cross-Sectional Design**

- **Reviewer’s Concern:** The reviewer objected to the persistence of causal language despite the study’s cross-sectional survey design.
- **Author’s Response:** We completely agree with this methodological critique. Given the cross-sectional nature of our survey, proving absolute causality is inappropriate.
- **Revision Made:** We have thoroughly audited the text and removed all definitive causal verbs (e.g., *causes, determines, proves*). They have been strictly replaced with association-based and predictive vocabulary (e.g., *influences, predicts, is associated with*) to align the prose with a cross-sectional framework.

**4. Theoretical Framework and Contemporary Research**

- **Reviewer’s Concern:** The framework was found to be largely descriptive with insufficient engagement with contemporary international research. Reference relevance and accuracy also require substantial correction.
- **Author’s Response:** This was an excellent recommendation. We have significantly updated the theoretical framework by integrating verified, highly relevant literature to ground our visual and behavioral constructs.
- **Revision Made:** We have integrated **5 genuine and highly cited international studies** into the literature framework under our sequential numbering style. These include empirical models on sales promotions and purchasing behaviors **(Mishra et al., 2024; 33)**, cognitive heuristics of charm pricing and brand perception **(Rashmi & Thomas, 2026; 34)**, the effects of price framing and magnitude imagery on perceived fairness **(Alnes & Haugom, 2024; 35)**, visual pricing cues and sensory marketing behavioral determinants **(Kheireddine & Saleh, 2025; 36)**, and strategic implementations of psychological pricing heuristics published under Springer Nature **(Alekri et al., 2026; 37)**. The section has been transformed from a descriptive layout into a critical, comparative discussion that directly supports our empirical findings.

**5. Data Availability Statement Controversy**

- **Reviewer’s Concern:** The statement claimed "no new data was created" despite collecting original questionnaires, while underlying data remained unavailable.
- **Author’s Response:** We apologize for the administrative drafting confusion in the previous statement. Original questionnaire data were indeed collected; however, they cannot be publicly deposited due to severe field constraints and strict ethical promises made to local participants in Iraq.
- **Revision Made:** The **Data Availability Statement** has been entirely rewritten in the manuscript to transparently reflect the reality of our field barriers. Due to heightened local anxieties surrounding identity theft, written records, and data misuse within the research environment in Iraq, participants strictly refused written signatures and only provided **oral informed consent** conditioned upon absolute anonymity and the post-analysis destruction of raw response files. We have retained the corresponding author’s official NTU institutional email within the statement for institutional verification.

**6. Prose, Grammar, and References**

- **Reviewer’s Concern:** The reviewer found the prose repetitive, grammatically weak, and reference accuracy in need of correction.
- **Author’s Response:** We have taken strict measures to address this.
- **Revision Made:** The manuscript has undergone a rigorous academic line-editing and proofreading process to eliminate structural redundancy and streamline the prose. Furthermore, the entire bibliography has been cross-checked, corrected for typographical precision, formatted into the journal's sequential numbering style, and directly linked with active DOI URLs to ensure digital verifiability.

# Reviewer report: Reviewer response for version 1

**DOI:** 10.5256/f1000research.195470.r509240

**Published:** 2026-08-21

**Authors:** Munther Khuder Yaqoob

**Affiliations:** 1 Marketing Management, University of Mosul, Mosul, Nineveh Governorate, Iraq

Observations on the research titled "

 The Role of Psychological Pricing Strategy in Customer Purchasing Behavior"

 This research examines the role and impact of psychological pricing strategies on consumer purchasing behavior. These strategies clearly influence purchasing patterns, especially since price is a significant and sensitive factor for some members of society when making purchases. An individual's income is a crucial indicator of their needs and desires, ultimately leading to their purchasing decisions.

 The current research topic is particularly important given the diversity of products and brands, and the proliferation of online and physical shopping platforms. This translates into intense competition among producers and marketers, necessitating informed decision-making by consumers when shopping for these products or brands.

 The research findings demonstrate a significant correlation between psychological pricing and consumer purchasing behavior, as well as the impact of psychological pricing, in all its dimensions and strategies, on consumer purchasing behavior.

 1. The abstract does not clarify the research methodology employed (whether descriptive, quantitative, descriptive-analytical, or another approach), nor does it specify the main hypothesis upon which the research is based, or the number of questionnaires collected from the research sample that were deemed unusable for analysis.

 2. The research does not present previous studies related to the current research variables. It is suggested that studies addressing these variables be included, along with an explanation of their similarities and differences to the current research, to inform the presentation of the results.

 3. The introduction needs a more systematic and scientific explanation of the current research problem. It requires rewording.

 4. The research problem section should include a sub-question regarding the intensity of the respondents' responses to the research variables.

 5. The theoretical, epistemological, and applied significance of the research should be clearly stated in the significance section.

 6. The presentation of the psychological pricing axes should begin by explaining the concept of psychological pricing and relying on modern sources. A new section should then be presented outlining the importance of psychological pricing for organizations and customers. Following this, the objectives of organizations offering psychological pricing to customers should be presented.

 7. The presentation should explain how psychological pricing strategies were chosen by presenting the percentage of agreement among researchers regarding the mentioned dimensions and providing a scientific justification for their selection, ensuring they are appropriate to the research context and the studied field.

 8. The presentation of the purchasing behavior axes should begin by explaining the concept of purchasing behavior and relying on modern sources. A new section should then be presented outlining the importance of studying purchasing behavior for organizations and customers. A brief overview of the factors influencing the study of customer purchasing behavior should then be presented.

 9. Supporting evidence should be provided for the selection of the dimensions for studying purchasing behavior, as explained in the previous section concerning the dimensions or strategies of psychological pricing.

 10. The presentation should focus on highlighting the weak results that emerged from the statistical analysis and utilizing them in the conclusions. A recommendation should be offered to address or strengthen these weaknesses.

 11. Each conclusion should have a corresponding recommendation. It is noted that there are 7 conclusions and 9 recommendations. A balance should be achieved.

 12. Ensure that the list of sources matches the text of the research.

Is the work clearly and accurately presented and does it cite the current literature?

Yes

If applicable, is the statistical analysis and its interpretation appropriate?

Yes

Are all the source data underlying the results available to ensure full reproducibility?

Yes

Is the study design appropriate and is the work technically sound?

Yes

Are the conclusions drawn adequately supported by the results?

Partly

Are sufficient details of methods and analysis provided to allow replication by others?

Yes

Reviewer Expertise:

Marketing Management, Digital Marketing, E- Marketing, Customer Behavior, and Marketing Strategies and Brand.

I confirm that I have read this submission and believe that I have an appropriate level of expertise to confirm that it is of an acceptable scientific standard, however I have significant reservations, as outlined above.

# Response

**Published:** 2026-09-07

**Authors:** Sawsan Ibrahim Rajab

**Affiliations:** Marketing management technique, Northern Technical University / Kirkuk Polytechnic College, Kirkuk, Kirkuk, Iraq

**First Reviewer's Comments**

**Dear Professor**

**It was a true honor to have you as one of the reviewers for my research. I would like to express my sincere gratitude and appreciation for the valuable time and effort you dedicated to reviewing my work.**

**All the comments you provided were undoubtedly aimed at elevating and improving the quality of the research. Most of these comments have been addressed and incorporated, and they were immensely helpful and valuable**

**Wishing you continued health and wellness.**

**With thanks and appreciation.**

# Reviewer report: Reviewer response for version 1

**DOI:** 10.5256/f1000research.195470.r509239

**Published:** 2026-08-19

**Authors:** Ahmed Hammadi

**Affiliations:** 1 University of Fallujah, Fallujah, Iraq

1. Title

 The title is generally clear and relevant to the study topic. However, it should be reconsidered to better reflect the actual research design and analytical relationships examined. The use of the term **“Role”** is somewhat vague, while the study actually examines correlation and statistical effects. A more precise title should identify the specific relationship between psychological pricing and consumer purchasing behavior and, if appropriate, the empirical context.

 2. Abstract

 The abstract provides a general overview of the study and reports the main findings. However, it needs substantial improvement in terms of scientific precision. The research gap, sampling technique, measurement procedure, and statistical methodology are not sufficiently explained. The abstract also appears to overstate the causal implications of the findings. Since the study is based on cross-sectional questionnaire data, causal expressions should be used cautiously.

 3. Introduction

 The introduction establishes the general relevance of psychological pricing and consumer behavior. Nevertheless, it is relatively descriptive and repetitive and does not establish a sufficiently convincing **research gap**. The authors should clearly explain what is unknown, inconsistent, or insufficiently examined in previous research and how the present study addresses that gap. The claim that the management of Al-Salam stores is not fully aware of the effects of psychological pricing appears to be based mainly on the researchers’ observations and requires stronger empirical or documentary support.

 4. Research Problem

 The research problem is clearly formulated; however, it remains largely **practical rather than theoretically driven**. Simply asking whether psychological pricing affects purchasing behavior is insufficient to establish a strong research problem for an international journal. The authors should formulate the problem around a clearly demonstrated gap in the existing literature, particularly conflicting findings, unexplored dimensions, contextual differences, or theoretical limitations.

 5. Research Objectives and Hypotheses

 The objectives and hypotheses are generally aligned with the research variables. However, the hypotheses are largely formulated as direct relationships without adequate theoretical justification. The use of the term **“effect”** is also problematic because the research design is cross-sectional and based on self-reported questionnaire data. The authors should either provide a stronger causal research design or use more appropriate terminology such as **“association”** or **“relationship.”**

 6. Theoretical Framework

 The theoretical framework is one of the major weaknesses of the manuscript. Although the study provides several definitions and descriptions of psychological pricing, the discussion remains largely **descriptive rather than explanatory**. The manuscript does not establish a strong theoretical mechanism explaining why and through what psychological processes pricing cues influence purchasing behavior.

 The authors should substantially strengthen the theoretical foundation by incorporating an appropriate consumer-behavior theory and explaining the mechanism connecting psychological pricing dimensions to cognitive, emotional, and behavioral responses. The framework should not simply list definitions and previous findings; it should develop a coherent theoretical argument leading to the proposed hypotheses.

 The previous review also identified weak theoretical integration and insufficient engagement with recent consumer-behavior literature.

 7. Literature Review

 The literature review requires considerable restructuring. It currently focuses heavily on definitions and general benefits of psychological pricing rather than critically synthesizing previous empirical studies. The authors should compare previous studies according to **context, variables, methodology, sample, findings, and limitations**, and then demonstrate precisely how the present study differs.

 Greater use of recent international literature, particularly studies published during **2024–2026**, is necessary. The existing literature base does not sufficiently demonstrate the novelty or international relevance of the study.

 8. Methodology

 The use of a descriptive-analytical approach and questionnaire survey is generally appropriate for an exploratory empirical study. However, the methodological description is not sufficiently detailed for replication.

 The authors should clarify:

- the exact sampling technique;
- the characteristics of the target population;
- the justification for the sample size;
- the criteria for inclusion and exclusion;
- the reasons for excluding the 112 questionnaires;
- the development and sources of questionnaire items;
- whether established measurement scales were adopted or developed;
- the pilot study procedure;
- procedures for content validity;
- reliability and construct validity testing.

The study states that 400 questionnaires were distributed and 288 valid questionnaires were analyzed, but the sampling process and selection procedure require substantially greater methodological clarification.

 9. Measurement Validity and Reliability

 This is a major methodological concern. The manuscript should provide sufficient evidence for the reliability and validity of the measurement model.

 In particular, the authors should report and interpret, where applicable:

- Cronbach’s alpha;
- Composite Reliability;
- Average Variance Extracted (AVE);
- convergent validity;
- discriminant validity;
- factor loadings;
- appropriate model-fit indices.

Simply reporting that a questionnaire was measured using a five-point Likert scale is not sufficient to establish measurement quality.

 10. Statistical Analysis

 The statistical analysis requires **careful re-examination**. The manuscript reports AMOS V24 results, including β, R², C.R., and p-values. However, several inconsistencies appear between the narrative and the statistical tables.

 For example, the terminology used for the pricing dimensions is inconsistent, including expressions such as **“fractional prices,” “decimal prices,” “price separator,” “price line size,” and “price font size.”** These inconsistencies make it difficult to determine whether the same constructs are being measured and tested throughout the manuscript.

 The authors should carefully audit all tables, variable labels, coefficients, statistical values, and corresponding textual interpretations. This issue is particularly important because statistical inconsistencies undermine confidence in the reported findings.

 11. SEM/AMOS Model

 If AMOS and structural equation modeling are being used, the manuscript should provide the complete measurement and structural model assessment. Reporting regression coefficients and p-values alone is insufficient.

 The authors should report appropriate **model-fit indices**, such as:

**χ²/df, CFI, TLI, RMSEA, SRMR**, and other relevant indicators, together with acceptable thresholds and interpretation.

 The manuscript should also distinguish clearly between the **measurement model** and the **structural model**.

 12. Extremely High Correlations and R²

 The reported relationships are unusually strong. For example, the overall correlation between psychological pricing and consumer purchasing behavior is reported as **0.91**, while R² is reported as **0.82**.

 Such high values require additional methodological justification. The authors should investigate the possibility of:

- conceptual overlap between constructs;
- common method bias;
- multicollinearity;
- insufficient discriminant validity;
- item redundancy.

The manuscript should provide appropriate diagnostic tests rather than assuming that the high coefficients automatically demonstrate a strong substantive effect.

 13. Results and Discussion

 The results are presented in relation to the hypotheses, which is useful. However, the discussion mainly **repeats the statistical findings** rather than critically interpreting them.

 The authors should explain:

- why the findings occurred;
- how they compare with previous studies;
- why they agree or disagree with previous findings;
- what theoretical implications arise from the results;
- what contextual factors in the Iraqi retail environment might explain the findings.

The discussion should therefore move beyond **“the hypothesis was accepted”** toward a deeper theoretical and empirical interpretation.

 14. Causality

 A major concern is the repeated use of causal language. The study uses a cross-sectional questionnaire and self-reported responses, which can demonstrate statistical associations but cannot, by themselves, establish strong causal relationships.

 Statements implying that psychological pricing **causes** purchasing behavior, increases sales, increases profits, or increases market share should therefore be substantially moderated unless direct longitudinal, experimental, or financial evidence is provided.

 15. Conclusions

 The conclusions generally correspond to the statistical findings, but some conclusions extend beyond what was actually measured.

 In particular, the manuscript links psychological pricing to **increased sales, profits, and market share**, although these outcomes were not directly measured in the study. This represents an important inferential leap. The conclusions should be restricted to the variables actually investigated.

 16. Recommendations

 The recommendations have practical relevance, but several are too general and repetitive. They should be more directly derived from the empirical findings.

 The authors should distinguish between recommendations supported directly by the results and suggestions requiring further empirical verification. For example, claims that psychological pricing increases organizational profits or market share should not be presented as established findings when financial or market-share data were not collected.

 17. References

 The reference list requires improvement in terms of **recency, consistency, and international coverage**. The authors should incorporate substantially more recent peer-reviewed studies and ensure that all references directly support the claims for which they are cited.

 The manuscript also needs consistent reference formatting and careful verification of bibliographic information.

 18. Language and Academic Writing

 The manuscript requires extensive English-language editing. There are repeated grammatical problems, awkward expressions, inconsistent terminology, unnecessary repetition, and instances that appear to reflect literal or mechanical translation.

 The terminology for the same constructs should be standardized throughout the manuscript. This issue was also explicitly identified in the previous peer-review report.

Is the work clearly and accurately presented and does it cite the current literature?

Yes

If applicable, is the statistical analysis and its interpretation appropriate?

Partly

Are all the source data underlying the results available to ensure full reproducibility?

Partly

Is the study design appropriate and is the work technically sound?

Yes

Are the conclusions drawn adequately supported by the results?

Partly

Are sufficient details of methods and analysis provided to allow replication by others?

Partly

Reviewer Expertise:

Marketing management, digital marketing, consumer behavior, strategic management, and management information technology.

I confirm that I have read this submission and believe that I have an appropriate level of expertise to confirm that it is of an acceptable scientific standard, however I have significant reservations, as outlined above.

# Response

**Published:** 2026-09-07

**Authors:** Sawsan Ibrahim Rajab

**Affiliations:** Marketing management technique, Northern Technical University / Kirkuk Polytechnic College, Kirkuk, Kirkuk, Iraq

**Dear Professor**

**It was a true honor to have you as one of the reviewers for my research. I would like to express my sincere gratitude and appreciation for the valuable time and effort you dedicated to reviewing my work.**

**All the comments you provided were undoubtedly aimed at elevating and improving the quality of the research. Most of these comments have been addressed and incorporated, and they were immensely helpful and valuable**

**Wishing you continued health and wellness.**

**With thanks and appreciation.**

# Reviewer report: Reviewer response for version 1

**DOI:** 10.5256/f1000research.195470.r495149

**Published:** 2026-06-24

**Authors:** George Spais

**Affiliations:** 1 Hellenic Open University, Patras, Greece

The manuscript uses a questionnaire survey of 288 respondents to examine whether psychological pricing strategies influence customer purchasing behavior in Kirkuk's AlSalam chain stores. Its stated goal is to contribute to retail pricing practice in a local market context by elucidating the relationship and effects of these pricing cues on cognitive, emotional, and purchase responses. The manuscript's contribution is limited by weak theoretical integration, inconsistent reporting, and insufficient engagement with recent F1000Research and 2025–2026 literature, despite the topic being fundamentally relevant to consumer behavior research. Here are the major strengths and weaknesses:

 Major strengths

 -Addresses a relevant consumer behavior topic with practical managerial interest in retail pricing.

 -Uses primary field data from an under-researched local retail context.

 -Attempts to examine multiple pricing cues rather than a single price-ending effect.

 Major weaknesses

 -Writing is repetitive, awkward, and often unprofessional, with clear signs of sloppiness and inconsistent terminology.

 -Tables and narrative contain inconsistencies, including variable-label confusion and questionable reporting of coefficients.

 -Theoretical positioning is weak and insufficiently connected to recent F1000Research and 2025–2026 studies.

 -Methodological reporting is incomplete.

 -References are uneven, stylistically mixed, and not always well aligned with in-text claims.

 -Ethics/data transparency is problematic.

 A. MAJOR CONCERNS  

  (1) The prose is often repetitive, generic, and awkwardly translated, with clear signs of sloppiness such as spacing/typing errors, inconsistent terminology, and chapter/sectioning confusion; examples include shifting labels for the same constructs (e.g., fractional/partial/sub-prices; price separators/price interval/decimal prices; price line/font size/color).

  (2) Additionally, the narrative and tables exhibit internal inconsistencies. Table 3 appears inconsistent with the text; some coefficients are duplicated or assigned to the wrong predictors, and "decimal prices" appear unexpectedly. The text, which requires significant editorial rewriting and clarification of authorship, exhibits formulaic phrasing and mechanical repetition.

  (3) The work is insufficiently positioned in the contemporary consumer-behavior literature, lacking a clear explanatory framework and showing limited engagement with recent F1000Research standards, where stronger theoretical integration and transparent evidence synthesis are expected. It also does not convincingly connect with newer 2025–2026 research on price endings, cultural variation, and quality/image inferences from pricing cues.

  (4) The work relies on a single-chain, cross-sectional, self-report survey, yet repeatedly uses causal language (“effect”); key details on measurement validity/reliability are missing.

  (5) Ethical and transparency concerns are serious as well, as the data availability statement says “no new data has been created”, although original questionnaire data were clearly collected, and the ethics/disclosure text is partly repetitive and unclear.

  (6) The references are mixed in style, uneven in completeness, and not always well aligned with in-text claims.

 B. OVERALL EVALUATION AND RECOMMENDATION

 You address a potentially relevant topic in consumer behavior and add practical relevance by examining psychological pricing cues in a local retail setting. However, in its current form, it falls well below a publishable standard.  The manuscript has weak, repetitive, and sloppy writing; inconsistent terminology; table-text mismatches; inadequate theoretical positioning; limited engagement with recent F1000Research and 2025–2026 literature; incomplete methodological and statistical reporting; and concerning ethics and data transparency issues. Since the manuscript would need significant redevelopment rather than a traditional major revision, I strongly recommend “rejection”.

Is the work clearly and accurately presented and does it cite the current literature?

Partly

If applicable, is the statistical analysis and its interpretation appropriate?

Partly

Are all the source data underlying the results available to ensure full reproducibility?

No

Is the study design appropriate and is the work technically sound?

Partly

Are the conclusions drawn adequately supported by the results?

Partly

Are sufficient details of methods and analysis provided to allow replication by others?

No

Reviewer Expertise:

promotion management, digital, promotions, digital marketing and marketing educations

I confirm that I have read this submission and believe that I have an appropriate level of expertise to state that I do not consider it to be of an acceptable scientific standard, for reasons outlined above.
